# Safety and Immunogenicity of an mRNA-Based RSV Vaccine Including a 12-Month Booster in a Phase 1 Clinical Trial in Healthy Older Adults

**DOI:** 10.1093/infdis/jiae081

**Published:** 2024-02-22

**Authors:** Christine A Shaw, Brandon Essink, Charles Harper, Runa Mithani, Archana Kapoor, Rakesh Dhar, Lauren Wilson, Ruiting Guo, Catherine A Panozzo, Eleanor Wilson, Alana K Simorellis, Caroline Reuter, Sonia K Stoszek, Grace L Chen, Rituparna Das, Jaya Goswami

**Affiliations:** Infectious Disease, Research and Development, Moderna, Inc, Cambridge, Massachusetts; Velocity Clinical Research, Omaha, Nebraska; Velocity Clinical Research, Omaha, Nebraska; Infectious Disease, Research and Development, Moderna, Inc, Cambridge, Massachusetts; Infectious Disease, Research and Development, Moderna, Inc, Cambridge, Massachusetts; Infectious Disease, Research and Development, Moderna, Inc, Cambridge, Massachusetts; Infectious Disease, Research and Development, Moderna, Inc, Cambridge, Massachusetts; Infectious Disease, Research and Development, Moderna, Inc, Cambridge, Massachusetts; Infectious Disease, Research and Development, Moderna, Inc, Cambridge, Massachusetts; Infectious Disease, Research and Development, Moderna, Inc, Cambridge, Massachusetts; Infectious Disease, Research and Development, Moderna, Inc, Cambridge, Massachusetts; Infectious Disease, Research and Development, Moderna, Inc, Cambridge, Massachusetts; Infectious Disease, Research and Development, Moderna, Inc, Cambridge, Massachusetts; Infectious Disease, Research and Development, Moderna, Inc, Cambridge, Massachusetts; Infectious Disease, Research and Development, Moderna, Inc, Cambridge, Massachusetts; Infectious Disease, Research and Development, Moderna, Inc, Cambridge, Massachusetts

**Keywords:** mRNA-1345, mRNA vaccine, older adult, respiratory syncytial virus, safety and immunogenicity

## Abstract

**Background:**

An mRNA-based respiratory syncytial virus (RSV) vaccine, mRNA-1345, is under clinical investigation to address RSV disease burden in older adults.

**Methods:**

Based on a randomized, observer-blind, placebo-controlled design, this phase 1 dose-ranging study evaluated the safety, reactogenicity, and immunogenicity of mRNA-1345 in adults aged 65 to 79 years. Participants were randomized to receive 1 dose of mRNA-1345 (12.5, 25, 50, 100, or 200 µg) or placebo and matched mRNA-1345 booster or placebo at 12 months.

**Results:**

Overall, 298 participants received the first injection and 247 received the 12-month booster injection. mRNA-1345 was generally well tolerated after both injections, with the most frequently reported solicited adverse reactions being injection site pain, fatigue, headache, arthralgia, and myalgia. Reactogenicity was higher after the booster injection but with severity, time to onset, and duration similar to the first injection. A single mRNA-1345 injection boosted RSV-A and RSV-B neutralizing antibody titers and prefusion F binding antibody (preF bAb) concentrations at 1 month (geometric mean fold rises: RSV-A, 10.2–16.5; RSV-B, 5.3–12.5; preF bAb, 7.2–12.1). RSV antibody levels remained above baseline through 12 months, indicating immune persistence. A 12-month booster injection also increased RSV-A and RSV-B neutralizing antibody titers and preF bAb concentrations; titers after booster injection were numerically lower than those after the first dose, with overlapping 95% CIs.

**Conclusions:**

mRNA-1345 was well tolerated and immunogenic following a single injection and a 12-month booster.

**Clinical Trials Registration:**

NCT04528719 (ClinicalTrials.gov).

## Teaser

Older adults and those with comorbidities are at increased risk of severe RSV. We assessed the safety and immunogenicity of mRNA-1345, an RSV vaccine, in adults aged 65 to 79 years. mRNA-1345 was well tolerated and immunogenic following a single injection and 12-month booster.

Respiratory syncytial virus (RSV) is a major cause of respiratory disease in persons of all ages globally [1–7]. Immunity following infection is typically short-lived [8–11]. Due to age-related decreases in immune function and the presence of underlying health conditions, older adults are at increased risk for serious RSV-associated complications, which can lead to hospitalizations and death [2, 4, 6, 12, 13]. Among adults aged ≥60 years in high-income countries, RSV caused an estimated 5.2 million cases of associated acute respiratory infection, leading to 470 000 hospitalizations and 33 000 in-hospital deaths in 2019 [14]. Furthermore, RSV incidence and impact in older adults are likely underestimated due to infrequent testing [15].

Treatment for RSV is supportive in older adults [2, 16], emphasizing the need for a safe and effective RSV vaccine to prevent RSV disease in this population. Improved understanding of the RSV envelope fusion (F) glycoprotein structure [17, 18] and the stabilization in the prefusion (preF) conformation has recently aided new vaccine development. The preF conformation is the preferred vaccine antigen as it exposes all known neutralizing epitopes in the protein and induces high neutralizing antibody (nAb) responses to the RSV-A and RSV-B subtypes [17, 19–22]. As of writing, 2 preF-based RSV vaccines are approved in the United States for the prevention of RSV in adults aged ≥60 years [23–27]. Several other RSV vaccines are in clinical development, including the novel RSV vaccine candidate mRNA-1345, an mRNA-based vaccine that encodes the membrane-anchored RSV F glycoprotein stabilized in the preF conformation and encapsulated in lipid nanoparticles (LNPs).

Based on a randomized, observer-blind, placebo-controlled design, this phase 1 dose-ranging study evaluated the safety, reactogenicity, and immunogenicity of the mRNA-1345 vaccine in healthy adults aged 65 to 79 years. This analysis supports the dose selected for the subsequent phase 2/3 pivotal efficacy trial of mRNA-1345 in older adults (ClinicalTrials.gov NCT05127434).

## METHODS

### Trial Design

This trial evaluated the safety, reactogenicity, and immunogenicity of a single injection of mRNA-1345 at 5 dose levels, as well as a booster injection given approximately 12 months after the first injection, in adults aged 65 to 79 years. This study is part of a larger ongoing phase 1 trial that includes women of childbearing potential aged 18 to 40 years, healthy adults aged 18 to 49 years, adults of Japanese descent aged ≥60 years, and children aged 12 to 59 months who are RSV seropositive. Here, we present data from the 65- to 79-year-old cohort through 14 months of follow-up; additional safety and immunogenicity results from future time points are anticipated.

The study was conducted in the United States (ClinicalTrials.gov, NCT04528719; Supplementary Methods). The protocol was approved by an institutional review board, and the trial was conducted according to the principles of the International Council for Harmonisation Technical Requirements for Registration of Pharmaceuticals for Human Use, the E6(R2) good clinical practice guidelines, and the Declaration of Helsinki, as well as all national, state, and local laws or regulations. All participants provided written informed consent for participation in the study.

### Trial Participants

Eligible participants were those who met the age criteria at the time of consent and were in good health based on review of medical history and screening physical examination (Supplementary Table 1). Full eligibility criteria are presented in the Supplementary Methods.

### Vaccine

mRNA-1345 contains a nucleoside-modified mRNA sequence encoding the membrane-anchored RSV F glycoprotein (RSV-A2 strain protein sequence) stabilized in the preF conformation through structural engineering and formulated in LNPs. The LNP formulation consists of an ionizable lipid, a phospholipid that forms lipid bilayer structures in LNPs, a polyethylene glycol lipid, and a sterol that improves stability [28, 29]. mRNA-1345 was provided as a lyophilized drug product and reconstituted with 0.9% sodium chloride to form a uniform 0.8-mg/mL mRNA-LNP dispersion; the vaccine was prepared for injection by diluting to yield a final volume of 0.5 mL for each dose level.

### Study Procedures

**Figure 1.**
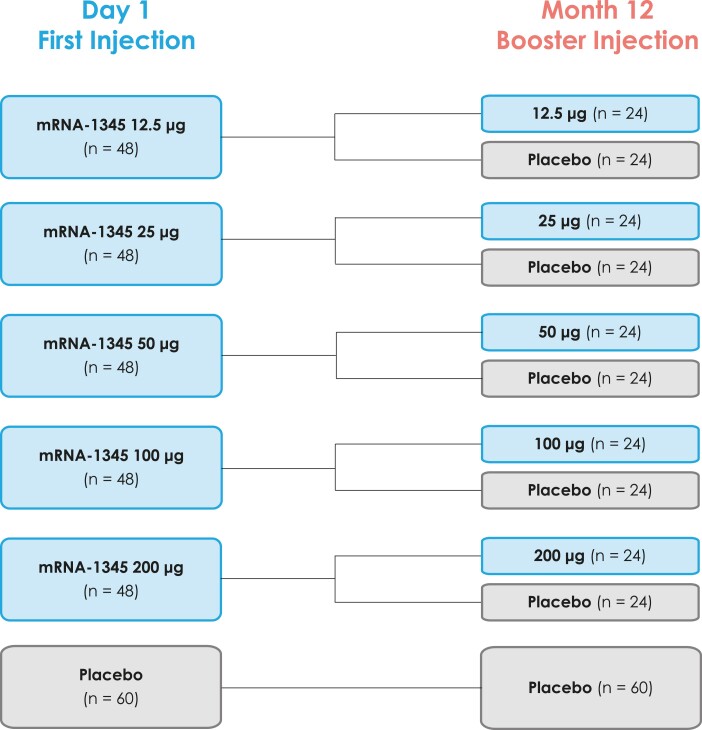
Schematic diagram of the planned treatment groups.

Participants were randomly assigned 2:2:1 via interactive response technology within each dose-level cohort (12.5, 25, 50, 100, or 200 μg; n = 60 each) to the following treatment sequences (day 1/month 12): mRNA-1345/mRNA-1345 (n = 24), mRNA-1345/placebo (n = 24), or placebo/placebo (n = 12; Figure 1). One dose of study vaccine (0.5 mL of mRNA-1345 or placebo [0.9% sodium chloride]) was administered by intramuscular injection into the deltoid muscle on day 1 and month 12. Data through 14 months (2 months after the booster injection) are presented in this article.

### Safety and Reactogenicity Assessments

The primary objective was to evaluate the reactogenicity and safety of mRNA-1345. Reactogenicity was assessed by solicited local and systemic adverse reactions (ARs). Solicited local ARs were injection site pain, erythema, and swelling/induration; solicited systemic ARs were fever, headache, fatigue, myalgia, arthralgia, nausea-vomiting, axillary swelling or tenderness on the same side as the injection (classified as “lymphadenopathy” in tables/figures), and chills. ARs were recorded by the participants via an electronic diary for 7 days after each injection. Solicited ARs were graded 1 to 4; additional information is provided in the Supplementary Methods.

Safety assessments consisted of unsolicited adverse events (AEs), serious AEs (SAEs), medically attended AEs (MAAEs), and AEs of special interest (AESIs; Supplementary Table 2). Unsolicited treatment-emergent AEs (TEAEs) after the first injection include all TEAEs up to 28 days after the first injection as well as all SAEs, MAAEs, fatal TEAEs, and AESIs collected from the first injection up to the booster injection or to end of study if participants did not receive a booster injection. Unsolicited TEAEs after the booster injection comprised all TEAEs up to 28 days after the booster injection, plus all MAAEs and AESIs collected from the booster injection up to month 14, as well as all SAEs and fatal TEAEs collected from the booster injection until the database lock date (6 February 2023). AEs leading to withdrawal were followed until resolution of the event. Unsolicited AEs were graded as mild, moderate, or severe; additional information is presented in the Supplementary Methods.

### Immunogenicity Assessments

Secondary objectives of the study were to evaluate antibody response to each vaccine dose level over time. Blood samples for antibody-mediated immunogenicity were collected prior to vaccination on day 1 and 1, 2, 3, 6, and 12 months after the first injection, as well as 1 and 2 months after the 12-month booster injection. Immunogenicity was assessed by measuring serum nAb titers against RSV-A and RSV-B and binding antibody (bAb) concentrations against the preF and postF conformations of the RSV F protein.

Qualified microneutralization assays were used to measure RSV-A and RSV-B nAb titers (Supplementary Methods), and results are presented as geometric mean titer (GMT; international units per milliliter [IU/mL] per World Health Organization international standard for antiserum to RSV) and geometric mean fold rise (GMFR; defined as the geometric mean ratio of postbaseline/baseline titers).

A qualified multiplex Luminex assay was used to measure preF and postF bAb (immunoglobulin G) concentrations (Supplementary Methods), and results are presented as geometric mean concentration (GMC; arbitrary units per milliliter) and GMFR.

### Statistical Analysis

There was no formal hypothesis testing. Three hundred older adults were planned to be enrolled in the study (240 mRNA-1345 recipients and 60 placebo recipients); this sample size was considered sufficient to provide a descriptive summary of the reactogenicity and immunogenicity of different dose levels of mRNA-1345 in this population. With 240 older adult participants receiving the investigational vaccine, there is at least a 95% probability to observe at least 1 participant with an AE at a true 1.24% AE rate.

Demographic variables and baseline characteristics were descriptively summarized by randomization group and overall study population: mean and SD for continuous variables and number and percentage for categorical variables. Safety analyses were descriptive and presented by treatment group. Immunogenicity analyses through 12 months were performed on the per-protocol set. Immunogenicity analyses through 14 months were performed on the per-protocol booster subset that received a first injection and a 12-month booster injection. Immunogenicity data were summarized at each time point, including GMT (nAbs), GMC (bAbs), and GMFR. The corresponding 95% CI was based on the *t* distribution of the log-transformed value and then back-transformed to the original scale. Analyses were performed with SAS version 9.4 (SAS Institute). Further information on the analysis populations is found in the Supplementary Methods.

## RESULTS

### Participants

A total of 300 participants aged 65 to 79 years were randomly assigned to receive a first injection of mRNA-1345 at 12.5, 25, 50, 100, or 200 μg (48 participants per dose level) or a placebo (60 participants; Figure 1, Supplementary Figure 1). Overall, 298 participants received the first injection, of which 239 received mRNA-1345 and 59 received placebo. Among these participants, 247 received a booster injection approximately 12 months later: 99 in the mRNA-1345/mRNA-1345 groups, 96 in the mRNA-1345/placebo groups, and 52 in the placebo/placebo group.

Between the first and booster (month 12) injections, 11.7% of participants in the placebo group and 17.1% in the mRNA-1345 groups discontinued study vaccination (Supplementary Figure 1). The most common reasons for vaccination discontinuation were withdrawal of consent (7.5% vaccine, 5.0% placebo) and loss to follow-up (5.0% vaccine, 6.7% placebo).

Between the booster injection and month 14, 3.8% of participants who received placebo/placebo, 8.3% of those who received mRNA-1345/placebo, and 5.1% of those who received mRNA-1345/mRNA-1345 discontinued the study (Supplementary Figure 1).

Demographics were generally well balanced across the first injection vaccination groups (Table 1). Among the participants in the mRNA-1345 dose groups, the median age ranged from 69.0 to 71.0 years, 52.1% to 60.4% were female, and 87.5% to 95.8% were White. Among the participants in the placebo group, the median age was 69.0 years, 55.0% were female, and 83.3% were White. Among the participants who received a booster injection, demographic data were similar to those who received a first injection (Supplementary Table 3).

**Table 1.** Baseline Demographics of Participants Receiving the First Injection: Randomized Set

		mRNA-1345^a^					
	Placebo (n = 60)^a^	12.5 µg (n = 48)	25 µg (n = 48)	50 µg (n = 48)	100 µg (n = 48)	200 µg (n = 48)	Total (n = 240)
Age, y, median	69.0	69.0	71.0	69.0	69.0	69.0	69.0
Sex^b^							
Female	33 (55.0)	29 (60.4)	26 (54.2)	25 (52.1)	25 (52.1)	28 (58.3)	133 (55.4)
Male	27 (45.0)	19 (39.6)	22 (45.8)	23 (47.9)	23 (47.9)	20 (41.7)	107 (44.6)
Race^b^							
White	50 (83.3)	44 (91.7)	43 (89.6)	45 (93.8)	42 (87.5)	46 (95.8)	220 (91.7)
Black or African American	7 (11.7)	4 (8.3)	2 (4.2)	1 (2.1)	6 (12.5)	1 (2.1)	14 (5.8)
Asian	1 (1.7)	0	2 (4.2)	1 (2.1)	0	0	3 (1.3)
American Indian or Alaska Native	0	0	0	0	0	1 (2.1)	1 (0.4)
Native Hawaiian or other Pacific Islander	1 (1.7)	0	0	0	0	0	0
Multiple	0	0	0	1 (2.1)	0	0	1 (0.4)
Other	1 (1.7)	0	1 (2.1)	0	0	0	1 (0.4)
Ethnicity^b^							
Hispanic or Latino	4 (6.7)	2 (4.2)	2 (4.2)	3 (6.3)	0	4 (8.3)	11 (4.6)
Not Hispanic or Latino	56 (93.3)	44 (91.7)	46 (95.8)	45 (93.8)	48 (100.0)	44 (91.7)	227 (94.6)
Not reported	0	2 (4.2)	0	0	0	0	2 (0.8)
BMI, kg/m^2^, mean ± SD	28.0 ± 3.9	28.8 ± 3.5	26.9 ± 4.1	27.8 ± 3.3	27.9 ± 3.9	26.9 ± 3.9	27.7 ± 3.8

Data are presented as No. (%) unless indicated otherwise.

Abbreviation: BMI, body mass index.

^a^Sample size per group indicates randomly assigned participants.

^b^Number indicates randomly assigned participants in the category with nonmissing data.

### Safety

#### Solicited ARs

**Figure 2.**
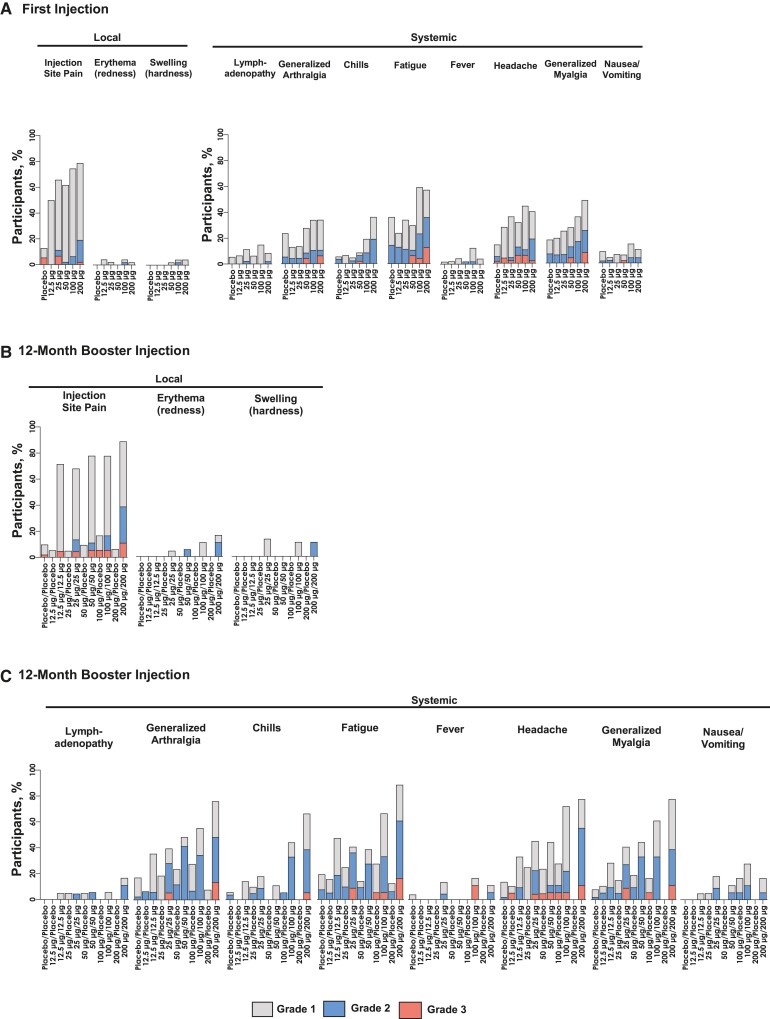
Solicited local and systemic adverse reactions within 7 days of receiving the first and booster injections. Number of participants per group after the first injection: placebo, n = 58; mRNA-1345, 12.5 µg, n = 46; 25 µg, n = 48; 50 µg, n = 47; 100 µg, n = 48; 200 µg, n = 47. Number of participants per group after booster injection: placebo/placebo, n = 51; mRNA-1345, 12.5 µg/placebo, n = 19; 12.5 µg/12.5 µg, n = 21; 25 µg/placebo, n = 20; 25 µg/25 µg, n = 22; 50 µg/placebo, n = 21; 50 µg/50 µg, n = 18; 100 µg/placebo, n = 18; 100 µg/100 µg, n = 18; 200 µg/placebo, n = 16; 200 µg/200 µg, n = 20.

The incidence of solicited local and systemic ARs after 1 injection of mRNA-1345 was lower in the mRNA-1345 groups receiving 12.5, 25, and 50 µg than 100 and 200 µg (Figure 2*A*, Supplementary Table 4).

The most common solicited local AR was injection site pain, which was reported in 50.0%, 65.9%, 61.7%, 74.5%, and 78.7% of participants in the mRNA-1345 groups receiving 12.5, 25, 50, 100, and 200 µg, respectively, and 12.7% of participants in the placebo group (Figure 2*A*, Supplementary Table 4).

The incidence of solicited systemic ARs after the first mRNA-1345 dose was approximately 50% in the groups receiving 12.5, 25, and 50 μg as compared with 78.7% and 66.0% in the groups receiving 100 and 200 μg, respectively, and 45.5% in the placebo group (Figure 2*A*). The most frequent solicited systemic ARs in the mRNA-1345 groups were fatigue, headache, myalgia, and arthralgia, with the highest rates in the 100- and 200-μg groups (Supplementary Table 4).

Most solicited ARs were grade 1 or 2 in severity, and grade 3 solicited ARs were more common in the higher mRNA-1345 dose groups; no grade 4 events were reported after the first injection.

The median duration of solicited local and systemic ARs overall in the mRNA-1345 groups was 1.0 to 2.0 days; the median duration of fatigue was slightly longer than the durations for other solicited systemic ARs, ranging from 1.5 days in the 50-μg group to 3 days in the 12.5-μg group.

Among those participants who received mRNA-1345 for their first injection, a higher frequency of solicited ARs was reported after a 12-month booster of mRNA-1345 vs after a 12-month injection of placebo (Figure 2*B* and 2*C*, Supplementary Table 5). At a matched mRNA-1345 dose level, the severity, time to onset, and duration of ARs were similar after the first and booster mRNA-1345 injections. One grade 4 event of myalgia occurred after the booster injection of 100-μg mRNA-1345.

#### Unsolicited AEs

After the first injection and up to the booster injection, no dose-dependent trends were observed in the incidence of unsolicited TEAEs, SAEs, MAAEs, or grade ≥3 events (regardless of causality) or in the incidence of unsolicited TEAEs considered injection related by the investigator. Unsolicited TEAEs, regardless of causality, were reported in 35.6%, 66.7%, 50.0%, 63.8%, 41.7%, and 60.4% of participants in the placebo and mRNA-1345 groups receiving 12.5, 25, 50, 100, and 200 µg, respectively (Supplementary Table 6). Unsolicited treatment-related TEAEs occurred in 10.4%, 4.2%, 4.3%, 10.4%, and 10.4% of participants in the groups receiving 12.5, 25, 50, 100, and 200 µg. Most events considered treatment related by the investigator were solicited AR terms or similar to solicited AR terms or were identified in 1 or 2 participants.

Regardless of causality, SAEs—as collected from the first injection up to the booster injection (month 12) or to study end if participants did not receive the booster injection—were reported in 5.9% of participants receiving mRNA-1345. There were no SAEs within 28 days of injection, and no SAEs were considered injection related per the investigator.

Review of all unsolicited TEAEs up to the booster injection or to study end if participants did not receive the booster injection did not identify any reports of anaphylactic reaction associated with mRNA-1345 administration, myocarditis/pericarditis, Guillain-Barré syndrome, Bell palsy/facial paralysis, acute demyelinating encephalomyelitis, seizures, or thrombocytopenia following receipt of mRNA-1345 (Supplementary Tables 2 and 6). No deaths, SAEs considered injection related per the investigator, or unsolicited TEAEs leading to study discontinuation were reported.

Across all cohorts in this study, 1 participant in the mRNA-1345 12.5-μg/placebo group discontinued the study vaccination due to an SAE unrelated to the study injection (gunshot wound).

After the 12-month booster injection through 14 months, unsolicited TEAEs were reported by 26.3%, 27.1%, and 19.2% of participants in the mRNA-1345/mRNA-1345 total group, the mRNA-1345/placebo total group, and the placebo/placebo group, respectively (Supplementary Table 7). Unsolicited treatment-related TEAEs after the 12-month booster injection occurred in 5.1%, 3.1%, and 3.8% of participants in the mRNA-1345/mRNA-1345 total group, the mRNA-1345/placebo total group, and the placebo/placebo groups. All events considered treatment related by the investigator were solicited AR terms or similar to solicited AR terms. SAEs were reported by 3.0%, 4.2%, and 1.9% of participants in the mRNA-1345/mRNA-1345 total group, the mRNA-1345/placebo total group, and the placebo/placebo group. No dose- or regimen-dependent trends were observed across individual dose levels in the mRNA-1345/mRNA-1345 or mRNA-1345/placebo total groups in the incidence of TEAEs, MAAEs, or SAEs after the 12-month booster injection. No TEAEs leading to study discontinuation were noted. No AESIs were reported, including anaphylactic reactions associated with vaccine administration, myocarditis/pericarditis, Guillain-Barré syndrome, acute disseminated encephalomyelitis, Bell palsy/facial paralysis, seizures, or thrombocytopenia.

During the booster follow-up period, 2 fatal events were reported: both were in participants who received a placebo dose as a booster injection, and both were considered unrelated to the study injection per the investigator. A recipient of mRNA-1345 12.5 μg/placebo died due to bone sarcoma, and a recipient of mRNA-1345 25 μg/placebo died due to a road traffic accident; both deaths occurred >28 days after the 12-month placebo injection and >1 year after the first mRNA-1345 injection.

### Immunogenicity

#### nAb and bAb Responses

**Figure 3.**
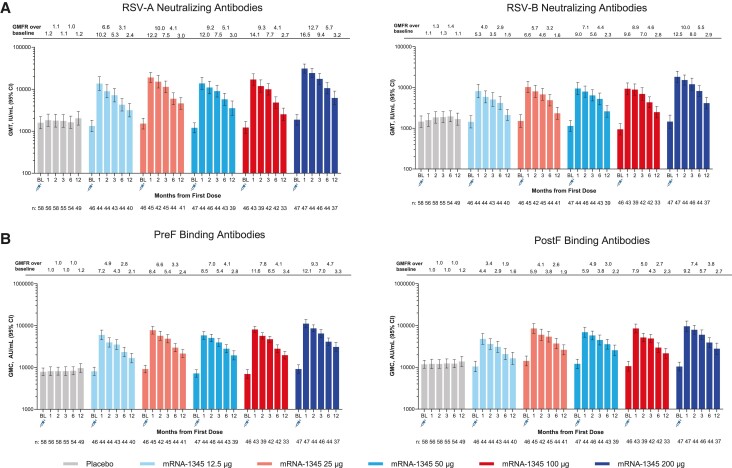
*A*, RSV-A and RSV-B neutralizing antibody GMTs and GMFR. *B*, preF and postF binding GMCs by time after the first injection (per-protocol set). GMC, geometric mean concentration; GMFR, geometric mean fold rise; GMT, geometric mean titer; postF, post–fusion F; preF, pre–fusion F; RSV, respiratory syncytial virus.

The baseline nAb GMTs and bAb GMCs across treatment groups were generally comparable between the mRNA-1345 dose groups and placebo group, with overlapping 95% CIs, and were consistent with prior exposure to RSV (Figure 3*A*, Supplementary Table 8).

A single mRNA-1345 injection elicited nAb responses against RSV-A and RSV-B subtypes at all dose levels evaluated. At 1 month after vaccination, RSV-A and RSV-B nAb GMTs were generally similar among the 25-, 50-, and 100-μg mRNA-1345 dose groups (RSV-A, 13 739.0–19 008.4; RSV-B, 9319.9-10 235.2), numerically lower in the 12.5-μg dose group (RSV-A, 13 619.5; RSV-B, 8154.1), and numerically higher in the 200-μg dose group (RSV-A, 31 084.4; RSV-B, 18 183.8). A similar trend was observed for nAb GMFRs at month 1. Across mRNA-1345 dose groups, the nAb GMFR from baseline for all mRNA-1345 dose groups was ≥10.2 for RSV-A and ≥5.3 for RSV-B.

The RSV-A and RSV-B nAb GMTs remained above baseline through 12 months after a single injection for all mRNA-1345 dose groups (GMFR ≥2.39 for RSV-A and ≥1.52 for RSV-B), demonstrating a persistence of immune response.

Similar trends were observed with bAbs (Figure 3*B*, Supplementary Table 9). At 1 month after the first vaccination, the bAb GMCs were similar among all mRNA-1345 dose groups but numerically higher in the 200-μg dose group. The preF bAb GMFR from baseline for all mRNA-1345 dose groups was ≥7.21 and remained above baseline through 12 months postdose (GMFR >2.0).

Although postF bAbs immune responses were similar to preF bAbs, the preF GMFR was greater than the postF GMFR at all time points after mRNA-1345 injection (Figure 3*B*).

#### nAb and bAb Response Following the Second Injection

**Figure 4.**
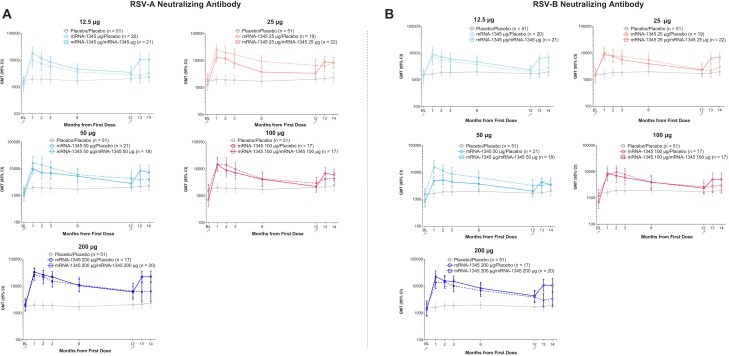
Serum RSV-A (*A*) and RSV-B (*B*) neutralizing antibody kinetics at baseline following the first injection and booster injection (per-protocol booster subset). GMT, geometric mean titer; RSV, respiratory syncytial virus.

After a booster injection of mRNA-1345 vaccination, administered approximately 12 months after the first mRNA-1345 vaccination, RSV nAb titers increased to levels similar to, albeit numerically lower than, those achieved after the first vaccination and with overlapping 95% CIs (Figure 4, Supplementary Table 10). Across mRNA-1345/mRNA-1345 dose groups, at 1 month after the first injection, the RSV nAb for GMFR from baseline ranged from 10.0 to 20.9 for RSV-A and 5.5 to 16.80 for RSV-B. By month 12 (before the booster injection), the GMFRs had declined to 2.4 to 3.2 for RSV-A and 1.5 to 3.4 for RSV-B. At 1 month after the booster mRNA-1345 injection (month 13), the nAb GMFRs from baseline (day 1) increased to 7.3 to 11.7 for RSV-A and 4.2 to 8.6 for RSV-B.

Similar to observations following the first injection, preF and postF bAbs GMCs also increased after a booster injection of mRNA-1345, and the preF bias was maintained (Supplementary Table 11).

## CONCLUSION

A phase 1 study was conducted to assess the reactogenicity, safety, and immunogenicity of mRNA-1345, an LNP-encapsulated mRNA-based vaccine encoding the membrane-anchored RSV F glycoprotein, in adults aged 65 to 79 years. mRNA-1345 produced a robust immune response across the dose levels evaluated and was well tolerated, and no safety concerns were identified after the first injection or the booster (month 12). The 50-μg mRNA-1345 dose level was selected for the subsequent phase 2/3 pivotal efficacy trial of mRNA-1345 in older adults (ClinicalTrials.gov NCT05127434) based on the acceptable safety and tolerability profile and for the generation of an optimal immune response in an immunosenescent population [30].

The frequency of local ARs after a single dose of mRNA-1345 was higher in the groups that received ≥100 μg of mRNA-1345. The most frequent local AR was pain at the injection site, and the most frequent solicited systemic ARs were headache, fatigue, myalgia, and arthralgia. The frequency of reactogenicity was higher after the booster injection (month 12) than after the first mRNA-1345 injection.

A single mRNA-1345 vaccination boosted RSV-A and RSV-B nAb titers as well as RSV preF bAb concentrations at all dose levels evaluated. Antibody responses against both RSV subtypes and with a preF bias were expected, given that the mRNA-1345–encoded preF protein is highly conserved across RSV subtypes and is stabilized in the preF conformation. The preF conformation is the main antigenic target of humoral immunity to RSV [31]. nAb titers after a single mRNA-1345 vaccination slowly declined through 12 months but were maintained above baseline, demonstrating durability of the immune response. A booster injection of mRNA-1345 administered approximately 12 months after the first injection boosted RSV nAb titers to within approximately 2-fold of those after the first dose; the titers after the booster injection were numerically lower, although the 95% CIs overlapped. Similar trends were observed at all dose levels. This is in line with the findings from a 12-month booster injection of 2 RSV preF subunit vaccines [32, 33].

A strength of the phase 1 trial is the randomized, observer-blind, placebo-controlled, dose-ranging study design. A study limitation is the limited sample sizes across individual treatment groups and the booster groups. Although no correlate of protection for RSV has been established, an immune response was demonstrated against all doses, and efficacy of the 50-µg dose of mRNA-1345 has been demonstrated in an ongoing phase 2/3 trial in adults aged ≥60 years [30]. In addition, although the current analysis in older adults was balanced across gender, there was a higher percentage of White participants than other races in the treatment groups. However, the ongoing phase 2/3 trial enrolled a globally diverse population across a range of age groups and risk factors and was broadly representative across race. Combined, the analysis of the phase 1 and 2/3 trials will allow for generalization of the reactogenicity, safety, and immunogenicity of mRNA-1345 across race and gender.

This phase 1 trial demonstrated that the mRNA-1345 vaccine is well tolerated and immunogenic in adults aged 65 to 79 years after a single injection and a 12-month booster injection. These results support the ongoing development of the mRNA-1345 vaccine in older adults.

## Supplementary Data

Supplementary materials are available at *The Journal of Infectious Diseases* online (http://jid.oxfordjournals.org/). Supplementary materials consist of data provided by the author that are published to benefit the reader. The posted materials are not copyedited. The contents of all supplementary data are the sole responsibility of the authors. Questions or messages regarding errors should be addressed to the author.

## Supplementary Material

jiae081_Supplementary_Data

## Notes

***Acknowledgments.*** We thank the participants in the trial and the members of the phase 1 trial team for their dedication and contributions to the trial, as well as the members of the data safety monitoring board for their hard work, support, and guidance of the trial. We acknowledge the contribution of Barbara J. Kuter, PhD, MPH, for critical reading of the manuscript. Medical writing and editorial assistance were provided by Jared Mackenzie, PhD, and Louansha Nandlal, PhD, of MEDiSTRAVA, in accordance with GPP3 guidelines (Good Publication Practice), funded by Moderna, Inc, and under the direction of the authors.

***Author contributions.*** A. K. was involved in data collection. C. A. S., R. M., R. D., L. W., J. G., E. W., R. G., A. K. S., C. R., S. K. S., and G. L. C. analyzed or interpreted the data. All authors provided writing, review, or intellectual contributions and approved the final draft.

***Data-sharing statement.*** Upon request and subject to review, Moderna, Inc, will provide the data that support the findings of this study. Moderna, Inc, may also provide access to the related individual anonymized participant data, subject to certain criteria, conditions, and exceptions.

***Financial support*.** This work was supported by Moderna, Inc.
