# Supplementary material for: Safety and Immunogenicity of an mRNA-Based RSV Vaccine Including a 12-Month Booster in a Phase 1 Clinical Trial in Healthy Older Adults
Source: J Infect Dis. 2024 Feb 22;230(3):e647–56. doi: 10.1093/infdis/jiae081 (PMC11420773; doi:10.1093/infdis/jiae081)
Supplement: jiae081_Supplementary_Data [file jiae081_supplementary_data.zip › Shaw_JID_Supplementary Methods_Clean.pdf]

Supplementary Methods

Study Inclusion criteria

For inclusion in the study, each participant was required to meet the following criteria:

1. Older adults aged ≥65 to <80 years who, in the opinion of the Investigator, are in good health based on review of medical history and screening physical examination.
2. Are able and willing to comply with protocol-mandated follow-up, including all procedures following the investigators assessment.
3. Has provided written informed consent for participation in this study, including all evaluations and procedures as specified by this protocol.
4. Has a body mass index (BMI) from ≥18 kg/m^2 to ≤35 kg/m^2.
5. Female participants of non–child-bearing potential could be enrolled in the study. Non–child-bearing potential was defined as bilateral tubal ligation >1 year prior to screening, bilateral oophorectomy, hysterectomy, or menopause. A follicle-stimulating hormone level could be measured at the discretion of the Investigator to confirm menopausal status.

Study Exclusion criteria

Any of the following was regarded as a criterion for exclusion of a participant from the study:

1. Had screening laboratory values > Grade 1.
2. Was acutely ill or febrile (temperature ≥38.0°C/100.4°F, regardless of route) on the day of the first injection. A participant meeting either of these criteria could be rescheduled for enrolment if the event resolved within the screening window.
3. Had a history of a diagnosis or condition that could affect study assessment or compromise participant safety based on the investigator's judgement. This includes:
    - Congenital or acquired immunodeficiency, including HIV infection.
    - Chronic hepatitis or suspected active hepatitis.

- A bleeding disorder that is considered a contraindication to intramuscular injection or phlebotomy.
- Dermatologic conditions that could affect local solicited adverse reaction (AR) assessments.
- Any history of allergic or anaphylactic reactions following a vaccination that could require medical intervention.
- Autoimmune disease except for Hashimoto’s disease.

4. Receipt of:
    - Systemic immunosuppressants or immune-modifying drugs for >14 days in total within 6 months prior to the day of enrollment (for corticosteroids, ≥2 mg/kg/day or ≥20 mg/day prednisone equivalent if the participant weighs >10 kg). Topical tacrolimus is allowed if not used within 14 days prior to the day of enrollment. Participants may be rescheduled for enrollment if they no longer meet this criterion within the screening window. Inhaled, nasal, and topical steroids are allowed.
    - Intravenous blood products (eg. red cells, platelets, Ig) within 3 months prior to enrollment.
5. Had received or planned to receive any licensed or authorized vaccine, including COVID-19 vaccines, for ≤28 days prior to the first injection (day 1) or planned to receive a licensed vaccine within 28 days before or after any study vaccine injection, with the exception of licensed influenza vaccines, which may be received more than 14 days before or after any study vaccine injection. Nonstudy vaccinations should not be delayed.
6. Had donated ≥450 mL of blood products within 28 days of the screening visit.
7. Had participated in an interventional clinical trial within 28 days prior to the day of enrollment or planned to do so while enrolled in this study.

8. Had a family member or household contact who is an employee of the research center or otherwise involved with the conduct of the study.
9. Has a history of myocarditis, pericarditis, or myopericarditis.
10. Known history of poorly controlled hypertension (per determination of the investigator) or systolic blood pressure >160 mmHg at the screening visit.
11. Known history of hypotension or systolic blood pressure <85 mmHg at the screening visit.
12. Poorly controlled diabetes mellitus (per determination of the investigator).
13. Diagnosis of significant chronic pulmonary disease (per determination of the investigator) (eg, chronic obstructive pulmonary disease, asthma).
14. Significant chronic cardiovascular disease (per determination of the investigator).
15. Resides in a nursing home.
16. Anticipates the need for immunosuppressive treatment at any time during participation in the study.
17. Diagnosis of malignancy within the previous 10 years (excluding nonmelanoma skin cancer and cervical carcinoma in situ).

Study Blinding

The study was observer blinded, with blinding of investigators, site personnel, and sponsor such that only designated unblinded study personnel responsible for vaccine preparation, administration, and/or accountability had access to study treatment assignments. Participant safety was monitored by a blinded internal safety monitoring team and an unblinded independent data safety monitoring board.

Safety Assessments

Grading for solicited ARs was based on grading scales modified from the Toxicity Grading Scale for Healthy Adult and Adolescent Volunteers Enrolled in Preventive Vaccine Trials.

	Grade 0	Grade 1 (mild)	Grade 2 (moderate)	Grade 3 (severe)	Grade 4[a] (life-threatening)
Injection site pain	None	Does not interfere with activity	Repeated use of over-the-counter pain reliever >24 hours or interferes with activity	Any use of prescription pain reliever or prevents daily activity	Requires emergency room visit or hospitalization
Injection site erythema (redness)	<25 mm/<2.5 cm	25–50 mm/2.5–5 cm	51–100 mm/5.1–10 cm	>100 mm/>10 cm	Necrosis or exfoliative dermatitis
Injection site swelling/induration (hardness)	<25 mm/<2.5 cm	25–50 mm/2.5–5 cm	51–100 mm/5.1–10 cm	>100 mm/ >10 cm	Necrosis
Headache	None	No interference with activity	Requires repeated use of over-the-counter pain reliever for >24 hours or some interference with activity	Significant; requires any use of prescription pain reliever or prevents daily activity	Requires emergency room visit or hospitalization
Fatigue	None	No interference with activity	Some interference with activity	Significant; prevents daily activity	Requires emergency room visit or hospitalization
Myalgia (muscle aches throughout body)	None	No interference with activity	Some interference with activity	Significant; prevents daily activity	Requires emergency room visit or hospitalization
Arthralgia (joint aches in several joints)	None	No interference with activity	Some interference with activity	Significant; prevents daily activity	Requires emergency room visit or hospitalization

Nausea/vomiting	None	No interference with activity or 1–2 episodes/ 24 hours	Some interference with activity or >2 episodes/ 24 hours	Prevents daily activity, requires outpatient intravenous hydration	Requires emergency room visit or hospitalization for hypotensive shock
Chills	None	No interference with activity	Some interference with activity not requiring medical intervention	Prevents daily activity and requires medical intervention	Requires emergency room visit or hospitalization
Fever (oral)	<38.0°C<br><100.4°F	38.0–38.4°C<br>100.4–101.1°F	38.5–38.9°C<br>101.2–102.0°F	39.0–40.0°C<br>102.1–104.0°F	>40.0°C<br>>104.0°F
Lymphadenopathy (underarm swelling or tenderness on same side as injection)	None	No interference with activity	Requires repeated use of over-the-counter (non-narcotic) pain reliever >24 hours or some interference with activity	Requires any use of prescription (narcotic) pain reliever or prevents daily activity	Emergency room visit or hospitalization

Note: Events listed above but starting >7 days post-study injection will be recorded on the adverse event page of the case report form.

[a]Grading for Grade 4 events per Investigator assessment (with exception of fever).

Immunogenicity Assessments

The neutralization assays were conducted as described previously [1]. The strains used in the RSV-A and RSV-B neutralization assay were A2 and B18537, respectively. Briefly, a constant amount of virus was mixed with serial dilutions of serum samples as well as the controls and added to a monolayer of HEp-2 cells (ATCC CCL-23). Following an incubation period of 24 hours at 37 °C, cells were fixed and immunostained with a monoclonal antibody directed against the RSV F protein; this was followed by a fixing and staining with a horseradish peroxidase (HRP)-conjugated secondary antibody (Life Technologies) and TrueBlue peroxidase substrate (Seracare). Plates were scanned, and spot counts were determined via ImmunoSpot/BioSpot CTL software. These data were used to determine the dilution of serum that reduced the virus signal by 50%.

IgG antibodies to RSV preF and postF antigens in serum were detected using a qualified multiplexed assay which is based on Luminex technology in which antigen-specific serum antibodies bind directly to the epitopes on antigens covalently conjugated to 2 distinct Luminex MagPlex-C (Superparamagnetic Carboxylated xMAP) microspheres. The serum samples were then incubated with antigen coated microspheres and then read on a Bio-Plex 200 (or equivalent) instrument, which identifies the specific Luminex microspheres by their distinct red and infrared fluorescent dye spectral properties. The measured fluorescent signal of the R-Phycoerythrin-conjugated AffiniPure F(ab')2 fragment goat anti-human IgG, Fcγ, fragment-specific secondary antibody is directly proportional to the amount of antigen specific serum IgG antibodies present in the serum samples.

Sample Size and Analysis Populations

The solicited safety set consisted of all participants who were randomly assigned and received any study injection and contributed any solicited AR data via electronic diary within 7 days of injection; the safety set consists of all randomly assigned participants who received any study injection and was used for analysis of safety data except for the solicited ARs. The safety set for the first vaccination was used for analysis of safety (except for solicited ARs) prior to booster vaccination and consisted of all randomly assigned participants who received the first study injection. The safety set for the booster vaccination was used for analysis of safety (except for solicited ARs) after the booster vaccination and consisted of all randomly assigned participants who received both first and booster study injections. The full analysis set (FAS) consisted of participants who received any study injection and have baseline data and ≥1 post-injection assessment. The per-protocol set was the primary population for immunogenicity assessments, which consisted of all FAS participants who complied with the injection schedule and the timings of immunogenicity blood sampling and had no major protocol violations that impacted immune response. The per-protocol booster subset was used for the 14-month immunogenicity analyses and consisted of participants that received a first injection on day 1 and booster injection at month 12.

References

1. Zielinska E, Liu D, Wu HY, Quiroz J, Rappaport R, Yang DP. Development of an improved microneutralization assay for respiratory syncytial virus by automated plaque counting using imaging analysis. Virol J **2005**; 2:84.
