# Supplementary material for: Safety and Immunogenicity of an mRNA-Based RSV Vaccine Including a 12-Month Booster in a Phase 1 Clinical Trial in Healthy Older Adults
Source: J Infect Dis. 2024 Feb 22;230(3):e647–56. doi: 10.1093/infdis/jiae081 (PMC11420773; doi:10.1093/infdis/jiae081)
Supplement: jiae081_Supplementary_Data [file jiae081_supplementary_data.zip › Shaw_Supplementary_Figure1.docx]

# Supplementary Figure 1. Disposition of participants after the first and booster injections


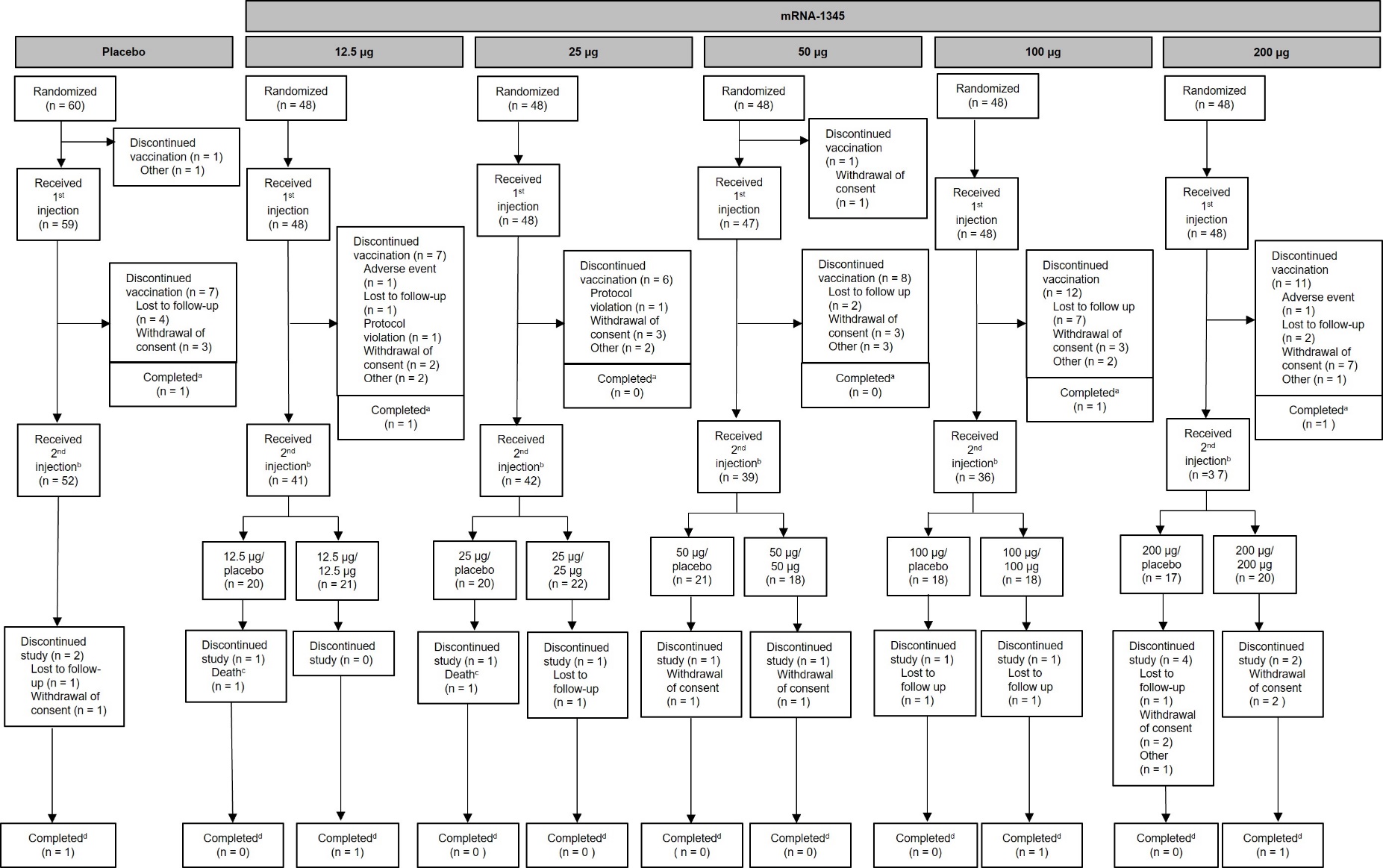


^a^Participants who completed the study at the time of data extraction. A participant who completes visits at least 6 months after the vaccination but no more than 12 months after vaccination but did not choose to enter the booster phase are considered to have completed the study.

^b^Participants were randomly assigned in a 2:2:1 ratio to day 1/month 12 sequences of mRNA-1345/mRNA-1345, mRNA-1345/placebo, or placebo/placebo.

^c^A recipient of mRNA-1345 12.5 μg/placebo died due to bone sarcoma and a recipient of mRNA-1345 25 μg/placebo died due to a road traffic accident; both deaths were reported >28 days after the 12-month placebo injection and >1 year after the first mRNA-1345 injection.

^d^Participants who completed visits at least 6 months after the last injection, and through an RSV season if this occurs later, but no more than 12 months after the last injection are considered to have completed the study.
