# Supplementary material for: Safety and Immunogenicity of an mRNA-Based RSV Vaccine Including a 12-Month Booster in a Phase 1 Clinical Trial in Healthy Older Adults
Source: J Infect Dis. 2024 Feb 22;230(3):e647–56. doi: 10.1093/infdis/jiae081 (PMC11420773; doi:10.1093/infdis/jiae081)
Supplement: jiae081_Supplementary_Data [file jiae081_supplementary_data.zip › Shaw_Supplementary_Table 1.docx]

**Table S1.** **Summary of Medical History by System Organ Class (Safety Set)**

|  | **Placebo** | **mRNA-1345** | | | | | |
| --- | --- | --- | --- | --- | --- | --- | --- |
|  | **(N = 59)^a^** | **12.5 µg (N = 48)^a^** | **25 µg (N = 48)^a^** | **50 µg (N = 47)^a^** | **100 µg (N = 48)^a^** | **200 µg (N = 48)^a^** | **Total (N = 239)^a^** |
| **n (%)^b^** |  |  |  |  |  |  |  |
| Number of Participants With at Least One Medical History | 59 (100.0) | 48 (100.0) | 48 (100.0) | 47 (100.0) | 48 (100.0) | 48 (100.0) | 239 (100.0) |
| Blood and lymphatic system disorders | 0 | 0 | 1 (2.1) | 4 (8.5) | 2 (4.2) | 0 | 7 (2.9) |
| Cardiac disorders | 5 (8.5) | 12 (25.0) | 7 (14.6) | 6 (12.8) | 2 (4.2) | 7 (14.6) | 34 (14.2) |
| Congenital, familial and genetic disorders | 2 (3.4) | 2 (4.2) | 0 | 2 (4.3) | 4 (8.3) | 1 (2.1) | 9 (3.8) |
| Ear and labyrinth disorders | 5 (8.5) | 5 (10.4) | 9 (18.8) | 5 (10.6) | 4 (8.3) | 4 (8.3) | 27 (11.3) |
| Endocrine disorders | 11 (18.6) | 6 (12.5) | 9 (18.8) | 4 (8.5) | 9 (18.8) | 9 (18.8) | 37 (15.5) |
| Eye disorders | 19 (32.2) | 15 (31.3) | 12 (25.0) | 17 (36.2) | 7 (14.6) | 7 (14.6) | 58 (24.3) |
| Gastrointestinal disorders | 24 (40.7) | 18 (37.5) | 25 (52.1) | 20 (42.6) | 24 (50.0) | 14 (29.2) | 101 (42.3) |
| General disorders and administration site conditions | 2 (3.4) | 4 (8.3) | 8 (16.7) | 3 (6.4) | 1 (2.1) | 2 (4.2) | 18 (7.5) |
| Hepatobiliary disorders | 8 (13.6) | 4 (8.3) | 5 (10.4) | 2 (4.3) | 4 (8.3) | 3 (6.3) | 18 (7.5) |
| Immune system disorders | 26 (44.1) | 24 (50.0) | 22 (45.8) | 22 (46.8) | 22 (45.8) | 24 (50.0) | 114 (47.7) |
| Infections and infestations | 11 (18.6) | 8 (16.7) | 9 (18.8) | 7 (14.9) | 6 (12.5) | 8 (16.7) | 38 (15.9) |
| Injury, poisoning and procedural complications | 8 (13.6) | 6 (12.5) | 9 (18.8) | 10 (21.3) | 1 (2.1) | 6 (12.5) | 32 (13.4) |
| Investigations | 6 (10.2) | 12 (25.0) | 2 (4.2) | 9 (19.1) | 4 (8.3) | 1 (2.1) | 28 (11.7) |
| Metabolism and nutrition disorders | 39 (66.1) | 32 (66.7) | 34 (70.8) | 28 (59.6) | 26 (54.2) | 21 (43.8) | 141 (59.0) |
| Musculoskeletal and connective tissue disorders | 22 (37.3) | 21 (43.8) | 29 (60.4) | 32 (68.1) | 26 (54.2) | 24 (50.0) | 132 (55.2) |
| Neoplasms benign, malignant and unspecified (incl cysts and polyps) | 12 (20.3) | 12 (25.0) | 8 (16.7) | 15 (31.9) | 12 (25.0) | 8 (16.7) | 55 (23.0) |
| Nervous system disorders | 9 (15.3) | 12 (25.0) | 14 (29.2) | 18 (38.3) | 14 (29.2) | 14 (29.2) | 72 (30.1) |
| Pregnancy, puerperium and perinatal conditions | 0 | 0 | 0 | 0 | 1 (2.1) | 0 | 1 (0.4) |
| Product issues | 0 | 0 | 1 (2.1) | 0 | 0 | 0 | 1 (0.4) |
| Psychiatric disorders | 20 (33.9) | 24 (50.0) | 13 (27.1) | 22 (46.8) | 21 (43.8) | 13 (27.1) | 93 (38.9) |
| Renal and urinary disorders | 6 (10.2) | 7 (14.6) | 8 (16.7) | 4 (8.5) | 4 (8.3) | 8 (16.7) | 31 (13.0) |
| Reproductive system and breast disorders | 18 (30.5) | 18 (37.5) | 11 (22.9) | 12 (25.5) | 12 (25.0) | 14 (29.2) | 67 (28.0) |
| Respiratory, thoracic and mediastinal disorders | 11 (18.6) | 7 (14.6) | 6 (12.5) | 8 (17.0) | 3 (6.3) | 4 (8.3) | 28 (11.7) |
| Skin and subcutaneous tissue disorders | 8 (13.6) | 4 (8.3) | 10 (20.8) | 5 (10.6) | 4 (8.3) | 3 (6.3) | 26 (10.9) |
| Social circumstances | 19 (32.2) | 16 (33.3) | 18 (37.5) | 22 (46.8) | 18 (37.5) | 22 (45.8) | 96 (40.2) |
| Surgical and medical procedures | 29 (49.2) | 25 (52.1) | 24 (50.0) | 25 (53.2) | 25 (52.1) | 19 (39.6) | 118 (49.4) |
| Vascular disorders | 28 (47.5) | 29 (60.4) | 31 (64.6) | 26 (55.3) | 20 (41.7) | 17 (35.4) | 123 (51.5) |

^a^Number of participants in the Safety Set who had this vaccination.

^b^Number of participants in the Safety Set who had this vaccination and reported the event.
