# Supplementary material for: Safety and Immunogenicity of an mRNA-Based RSV Vaccine Including a 12-Month Booster in a Phase 1 Clinical Trial in Healthy Older Adults
Source: J Infect Dis. 2024 Feb 22;230(3):e647–56. doi: 10.1093/infdis/jiae081 (PMC11420773; doi:10.1093/infdis/jiae081)
Supplement: jiae081_Supplementary_Data [file jiae081_supplementary_data.zip › Shaw_Supplementary_Table 10.docx]

**T****able S10. Neutralizing Antibody Titers After the Booster Injection (Per-protocol Booster Set)**

|  |  | **mRNA-1345/Placebo** | | | | | | | | **mRNA-1345/mRNA-1345** | | | | | | |
| --- | --- | --- | --- | --- | --- | --- | --- | --- | --- | --- | --- | --- | --- | --- | --- | --- |
|  | **Placebo/**  **Placebo** | **mRNA-1345**  **12.5 µg/**  **Placebo** | | **mRNA-1345**  **25 µg/**  **Placebo** | **mRNA-1345**  **50 µg/**  **Placebo** | | **mRNA-1345**  **100 µg/**  **Placebo** | **mRNA-1345**  **200 µg/**  **Placebo** | | **mRNA-1345**  **12.5 µg/**  **mRNA-1345  12.5 µg** | | **mRNA-1345**  **25 µg/**  **mRNA-1345 25 µg** | **mRNA-1345**  **50 µg/**  **mRNA-1345  50 µg** | **mRNA-1345**  **100 µg/**  **mRNA-1345  100 µg** | | **mRNA-1345**  **200 µg/**  **mRNA-1345**  **200 µg** |
|  | **n = 51^a^** | **n = 20^a^** | | **n = 19^a^** | **n = 21^a^** | | **n = 17^a^** | **n = 17^a^** | | **n = 21^a^** | | **n = 22^a^** | **n = 18^a^** | **n = 17^a^** | | **n = 20^a^** |
| **RSV-A Neutralization (IU/mL)** | | |  | | |  | | |  | |  | | | |  | |
| Baseline (day 1), n^b^ | 51 | 20 | | 19 | 21 | | 17 | 17 | | 21 | | 22 | 18 | 17 | | 20 |
| GMT  (95% CI) | 1702.2  (1200.4, 2413.7) | 1209.1  (703.3, 2078.8) | | 2037.9  (1306.7, 3178.0) | 1312.6  (871.6, 1976.7) | | 1481.4  (810.8, 2706.8) | 1870.8  (1175.1, 2978.3) | | 1416.4  (925.1, 2168.5) | | 1227.9  (782.7, 1926.5) | 1038.7  (623.1, 1731.4) | 695.6  (404.4, 1196.3) | | 2043.0  (1208.1, 3454.9) |
| Month 1  (day 29), n^c^ | 49 | 19 | | 19 | 19 | | 17 | 17 | | 21 | | 21 | 17 | 17 | | 20 |
| GMT  (95% CI) | 1978.7  (1388.5, 2819.8) | 10 007.5  (5436.0, 18 423.8) | | 25 714.6  (17 405.0, 37 991.4) | 15 825.0  (8877.6, 28 209.2) | | 14 817.7  (8043.1, 27 298.4) | 27 046.2  (16 297.3, 44 884.6) | | 18 307.1  (10 731.5, 31 230.4) | | 12 842.5  (8770.6, 18 804.9) | 9951.3  (5910.6, 16 754.4) | 14 526.5  (9010.1, 23 420.3) | | 33 624.6  (23 792.5, 47 519.9) |
| GMFR  (95% CI) | 1.2  (1.0, 1.4) | 7.9  (4.6, 13.7) | | 12.6  (7.9, 20.2) | 13.1  (8.4, 20.2) | | 10.0  (5.5, 18.1) | 14.5  (9.6, 21.9) | | 12.9  (7.5, 22.3) | | 10.0  (5.9, 16.8) | 10.1  (5.5, 18.5) | 20.9  (12.0, 36.2) | | 16.5  (9.9, 27.3) |
| Month 2  (day 57), n^c^ | 51 | 19 | | 17 | 21 | | 15 | 15 | | 20 | | 21 | 17 | 15 | | 19 |
| GMT  (95% CI) | 1915.0  (1322.9, 2772.0) | 7495.1  (4100.3, 13 700.7) | | 19 274.9  (10 629.3, 34 952.6) | 14 004.7  (7710.8, 25 436.2) | | 13 699.9  (7309.1, 25 678.5) | 21 643.6  (12 038.1,  38 913.6) | | 11 607.3  (6960.0, 19 357.7) | | 12 047.6  (7882.8, 18412.8) | 6964.2  (4141.7, 11 710.1) | 8737.9  (4897.5, 15 589.9) | | 26 141.3 (17 400.8, 39 272.1) |
| GMFR  (95% CI) | 1.1  (0.9, 1.4) | 6.1  (4.0, 9.4) | | 9.4  (5.2, 17.0) | 10.7  (6.6, 17.3) | | 8.8  (4.6, 17.0) | 11.1  (6.7, 18.5) | | 8.0  (5.1, 12.6) | | 10.1  (6.1, 16.6) | 6.9  (4.0, 11.8) | 11.1  (6.3, 19.5) | | 12.7  (7.7, 20.7) |
| Month 3 (day 85), n^c^ | 48 | 19 | | 19 | 19 | | 17 | 17 | | 20 | | 21 | 17 | 15 | | 19 |
| GMT  (95% CI) | 1889.4  (1302.5, 2740.9) | 6057.8  (3170.8, 11 573.2) | | 17 027.9  (10 453.7, 27 736.7) | 10 809.0  (6038.6, 19 347.9) | | 9813.6  (5674.5, 16 971.8) | 15 361.2  (9180.6, 25 703.0) | | 8432.7  (5164.8, 13 768.3) | | 7936.0  (5208.1, 12 092.7) | 6880.0  (4095.2, 11 558.6) | 7130.7  (4014.4, 12 666.2) | | 21 873.3  (13 748.1, 34 800.3) |
| GMFR  (95% CI) | 1.1  (1.0, 1.3) | 5.1  (3.1, 8.7) | | 8.4  (5.1, 13.8) | 8.2  (4.8, 13.9) | | 6.6  (4.2, 10.5) | 8.2  (5.3, 12.7) | | 5.8  (3.7, 9.2) | | 6.6  (4.1, 10.8) | 6.8  (4.0, 11.5) | 9.7  (5.6, 16.6) | | 10.6  (6.9, 16.2) |
| Month 6 (Day 169), n^c^ | 50 | 20 | | 19 | 20 | | 16 | 17 | | 21 | | 20 | 16 | 16 | | 18 |
| GMT  (95% CI) | 1705.9  (1194.0, 2437.4) | 3649.4  (2022.0, 6586.5) | | 9257.1  (5742.1, 14 923.9) | 5972.5  (3508.6, 10 166.7) | | 4185.8  (2233.4, 7844.9) | 11 153.5  (6072.0, 20 487.3) | | 4584.1  (2878.5, 7300.2) | | 3756.8  (2519.4, 5602.2) | 5224.3  (3003.0, 9088.7) | 4065.6  (2428.6, 6806.0) | | 10 481.3  (6217.5, 17 669.2) |
| GMFR  (95% CI) | 1.0  (0.9, 1.2) | 3.0  (2.0, 4.6) | | 4.5  (2.8, 7.4) | 4.8  (3.0, 7.6) | | 3.0  (1.5, 5.8) | 6.0  (4.0, 9.0) | | 3.2  (2.1, 5.0) | | 3.5  (2.2, 5.6) | 5.5  (3.3, 9.0) | 6.2  (3.9, 10.0) | | 5.2  (3.4, 7.8) |
| Month 12  (day 365), n^a^ | 49 | 20 | | 19 | 21 | | 16 | 17 | | 20 | | 22 | 18 | 17 | | 20 |
| GMT  (95% CI) | 2035.5  (1403.1, 2952.8) | 2874.3  (1691.0, 4885.8) | | 6590.4  (4013.5, 10 821.9) | 4320.6  (2265.8, 8238.8) | | 2932.2  (1748.2, 4917.9) | 6461.4  (3266.1, 12 782.9) | | 3477.2  (1990.3, 6074.7) | | 3361.7  (2262.5, 4995.0) | 2791.9  (1756.5, 4437.8) | 2194.7  (1339.1, 3596.9) | | 5988.2  (3801.6, 9432.6) |
| GMFR  (95% CI) | 1.2  (1.0, 1.4) | 2.4  (1.7, 3.4) | | 3.2  (2.0, 5.4) | 3.3  (2.0, 5.5) | | 2.2  (1.4, 3.4) | 3.5  (2.1, 5.6) | | 2.4  (1.6, 3.6) | | 2.7  (1.9, 3.9) | 2.7  (1.8, 4.0) | 3.2  (1.9, 5.2) | | 2.9  (2.0, 4.3) |
| Month 1 after booster  (day 393), n^c^ | 48 | 19 | | 17 | 21 | | 15 | 15 | | 21 | | 22 | 17 | 16 | | 17 |
| GMT  (95% CI) | 2131.5  (1436.7, 3162.4) | 2667.4  (1404.5, 5065.9) | | 6256.1  (3908.2, 10 014.7) | 3826.9  (2154.8, 6796.5) | | 4274.3  (2537.0, 7201.3) | 6103.9  (2601.7, 14 320.5) | | 10 415.0  (6287.6, 17 251.8) | | 8899.1  (5580.1, 14 192.2) | 8354.7  (5328.1, 13 100.4) | 6702.8  (3860.5, 11 637.7) | | 21 952.8  (13 309.8, 36 208.3) |
| GMFR  (95% CI) | 1.2  (1.0, 1.6) | 2.1  (1.4, 3.2) | | 3.1  (2.0, 5.0) | 2.9  (1.8, 4.7) | | 2.5  (1.6, 3.9) | 3.4  (1.8, 6.2) | | 7.4  (4.7, 11.4) | | 7.3  (4.2, 12.5) | 7.3  (4.3, 12.5) | 9.0  (5.7, 14.4) | | 11.7  (7.5, 18.3) |
| Month 2 after booster  (day 421), n^c^ | 46 | 18 | | 17 | 21 | | 15 | 14 | | 20 | | 21 | 14 | 15 | | 15 |
| GMT  (95% CI) | 2342.2  (1503.5, 3648.7) | 3242.9  (1633.9, 6436.5) | | 8061.5  (5101.2, 12 739.8) | 4005.7  (2125.0, 7550.7) | | 4394.9  (2576.9, 7495.6) | 6358.9  (2697.2, 14 991.8) | | 10 205.8  (5902.0, 17 647.9) | | 8406.9  (5416.1, 13 049.2) | 7240.7  (4089.7, 12 819.5) | 6121.3  (3796.9, 9868.6) | | 22 508.8  (13 906.0, 36 433.8) |
| GMFR  (95% CI) | 1.4  (1.1, 1.7) | 2.6  (1.7, 3.8) | | 4.0  (2.6, 6.2) | 3.1  (1.8, 5.1) | | 2.6  (1.7, 3.8) | 3.6  (2.1, 6.1) | | 7.4  (4.5, 12.2) | | 7.5  (4.4, 12.9) | 7.0  (4.4, 11.2) | 8.1  (5.4, 12.3) | | 9.5  (5.9, 15.3) |
| **RSV-B Neutralization (IU/mL)** | | | | | | | | | | | | | | | | |
| Baseline (day 1), n^b^ | 51 | 20 | | 19 | 21 | | 17 | 17 | | 21 | | 22 | 18 | 17 | | 20 |
| GMT  (95% CI) | 1507.5  (1086.3, 2092.2) | 1299.2  (693.3, 2434.6) | | 1396.9  (795.3, 2453.8) | 1541.7  (954.8, 2489.5) | | 1173.3  (647.6, 2125.8) | 1517.3  (825.2, 2790.1) | | 1509.4  (945.7, 2409.3) | | 1487.0  (841.3, 2628.3) | 806.1  (513.0, 1266.4) | 682.0  (389.9, 1192.9) | | 1328.7  (758.8, 2326.9) |
| Month 1  (day 29), n^c^ | 49 | 19 | | 19 | 19 | | 17 | 17 | | 21 | | 21 | 17 | 17 | | 20 |
| GMT  (95% CI) | 1615.0  (1102.5, 2365.8) | 7184.8  (3769.3, 13 694.9) | | 10 646.6  (6526.1, 17 368.7) | 15 750.1  (9278.5, 26 735.4) | | 7440.1  (4465.1, 12 397.4) | 13 528.3  (7893.1, 23 186.7) | | 9012.4  (4995.8, 16 258.3) | | 8730.9  (5268.5, 14 468.8) | 4770.2  (3117.9, 7297.9) | 8519.4  (4759.4, 15 250.0) | | 22 371.6  (13 621.6, 36 742.2) |
| GMFR  (95% CI) | 1.1  (1.0, 1.3) | 4.9  (2.7, 9.1) | | 7.6  (5.5, 10.6) | 11.3  (7.6, 16.7) | | 6.3  (4.0, 10.1) | 8.9  (5.5, 14.4) | | 6.0  (3.6, 9.8) | | 5.5  (3.2, 9.4) | 6.4  (3.9, 10.5) | 12.5  (8.1, 19.2) | | 16.8  (10.8, 26.3) |
| Month 2  (day 57), n^c^ | 51 | 19 | | 17 | 21 | | 15 | 15 | | 20 | | 21 | 17 | 15 | | 19 |
| GMT  (95% CI) | 1890.3  (1353.6, 2639.8) | 5574.3  (3049.3, 10 190.2) | | 7797.7  (4398.4, 13 824.1) | 11 106.4  (6629.5, 18 606.3) | | 9843.6  (5970.2, 16 230.0) | 13 876.6  (8313.3, 23 162.8) | | 7167.4  (4493.5, 11 432.5) | | 7437.0  (4649.0, 11 896.8) | 5141.0  (3444.0, 7674.3) | 7042.3  (3789.4, 13 087.5) | | 15 444.1  (9730.1, 24 513.8) |
| GMFR  (95% CI) | 1.3  (1.1, 1.5) | 4.2  (2.8, 6.4) | | 5.9  (3.9, 9.0) | 7.2  (4.6, 11.2) | | 8.3  (4.6, 14.7) | 8.2  (5.0, 13.5) | | 4.7  (3.2, 6.9) | | 5.2  (3.5, 7.9) | 6.8  (4.2, 11.1) | 9.1  (6.1, 13.6) | | 10.9  (7.0, 16.8) |
| Month 3 (day 85), n^c^ | 48 | 19 | | 19 | 19 | | 17 | 17 | | 20 | | 21 | 17 | 15 | | 19 |
| GMT  (95% CI) | 1876.2  (1355.4, 2597.2) | 4861.5  (2472.3, 9559.4) | | 7316.0  (4058.1, 13 189.2) | 8749.3  (5073.2, 15 089.0) | | 7748.0  (4570.0, 13 135.8) | 10 266.7  (6040.0, 17 451.1) | | 5957.9  (3513.6, 10 102.3) | | 5564.4  (3531.5, 8767.5) | 4376.7  (2848.8, 6724.0) | 5908.6  (3128.9, 11 157.9) | | 14 894.6  (9407.9, 23 581.3) |
| GMFR  (95% CI) | 1.2  (1.1, 1.4) | 3.7  (2.2, 6.1) | | 5.2  (3.5, 7.7) | 5.3  (3.3, 8.4) | | 6.6  (4.8, 9.0) | 6.8  (4.5, 10.3) | | 3.9  (2.5, 6.1) | | 3.9  (2.6, 5.9) | 5.8  (3.4, 9.9) | 8.5  (4.8, 15.0) | | 10.5  (7.2, 15.3) |
| Month 6 (day 169), n^c^ | 50 | 20 | | 19 | 20 | | 16 | 17 | | 21 | | 20 | 16 | 16 | | 18 |
| GMT  (95% CI) | 1966.2  (1412.7, 2736.7) | 3729.3  (2005.7, 6934.1) | | 5414.1  (3058.2, 9584.8) | 6391.1  (3844.0, 10 625.9) | | 3859.9  (2307.3, 6457.1) | 6819.8  (4130.1, 11 261.1) | | 4727.0  (2815.8, 7935.4) | | 3942.2  (2672.6, 5814.8) | 3683.3  (2296.2, 5908.3) | 3963.6  (2179.7, 7207.3) | | 8263.7  (4941.3, 13 819.9) |
| GMFR  (95% CI) | 1.3  (1.1, 1.5) | 2.9  (2.0, 4.2) | | 3.9  (2.7, 5.6) | 4.1  (2.8, 6.0) | | 3.5  (2.7, 4.6) | 4.5  (3.0, 6.7) | | 3.1  (2.1, 4.7) | | 2.6  (1.5, 4.6) | 4.5  (2.7, 7.3) | 6.1  (3.9, 9.5) | | 5.9  (4.3, 8.1) |
| Month 12  (day 365), n^c^ | 49 | 20 | | 19 | 21 | | 16 | 17 | | 20 | | 22 | 18 | 17 | | 20 |
| GMT  (95% CI) | 1673.2  (1190.9, 2350.8) | 1945.1  (1150.9, 3287.3) | | 2370.3  (1339.2, 4195.3) | 3253.8  (1951.5, 5425.2) | | 2661.3  (1607.4, 4406.4) | 3881.1  (2412.6, 6243.3) | | 2297.3  (1565.4, 3371.6) | | 2279.6  (1474.1, 3525.2) | 1984.9  (1331.4, 2959.1) | 2312.9  (1453.8, 3679.6) | | 4349.1  (2713.7, 6969.9) |
| GMFR  (95% CI) | 1.1  (0.9, 1.3) | 1.5  (1.1, 2.0) | | 1.7  (1.2, 2.4) | 2.1  (1.4, 3.1) | | 2.3  (1.7, 3.1) | 2.6  (1.8, 3.7) | | 1.5  (1.1, 2.2) | | 1.5  (1.1, 2.2) | 2.5  (1.8, 3.4) | 3.4  (2.1, 5.5) | | 3.3  (2.4, 4.4) |
| Month 1 after booster  (day 393), n^c^ | 48 | 19 | | 17 | 21 | | 15 | 15 | | 21 | | 22 | 17 | 16 | | 17 |
| GMT  (95% CI) | 1740.1  (1180.7, 2564.6) | 2391.6  (1279.1, 4471.7) | | 2142.3  (1198.9, 3828.1) | 3286.8  (1925.4, 5610.7) | | 2734.8  (1592.0, 4698.1) | 2987.2  (1654.6, 5392.9) | | 6330.1  (4382.2, 9143.9) | | 6224.7  (3932.9, 9852.2) | 4314.9  (3002.8, 6200.2) | 5144.6  (2943.2, 8992.4) | | 10 799.1  (6462.6, 18 045.7) |
| GMFR  (95% CI) | 1.1  (0.9, 1.4) | 2.0  (1.4, 3.0) | | 1.6  (1.2, 2.2) | 2.1  (1.6, 2.9) | | 2.1  (1.4, 3.1) | 2.0  (1.3, 3.1) | | 4.2  (2.7, 6.6) | | 4.2  (2.7, 6.5) | 5.2  (3.4, 7.9) | 7.2  (4.0, 12.8) | | 8.6  (4.8, 15.5) |
| Month 2 after booster  (day 421), n^c^ | 46 | 18 | | 17 | 21 | | 15 | 14 | | 20 | | 21 | 14 | 15 | | 15 |
| GMT  (95% CI) | 1983.8  (1330.2, 2958.7) | 2788.8  (1464.2, 5311.8) | | 3013.1  (1559.4, 5822.2) | 3584.5  (2150.4, 5975.0) | | 2969.5  (1619.6, 5444.7) | 3370.1  (1737.3, 6537.3) | | 6864.7  (4452.2, 10 584.3) | | 6837.6  (4515.8, 10 353.2) | 3431.2  (2298.0, 5123.1) | 5010.0  (2808.0, 8938.8) | | 10 588.5  (5841.4, 19 193.2) |
| GMFR  (95% CI) | 1.2  (1.0, 1.5) | 2.4  (1.5, 3.7) | | 2.3  (1.8, 2.9) | 2.3  (1.8, 3.1) | | 2.2  (1.6, 3.2) | 2.4  (1.6, 3.6) | | 4.5  (3.0, 6.7) | | 4.3  (2.7, 6.9) | 4.5  (2.8, 7.2) | 7.1  (4.4, 11.6) | | 8.3  (4.4, 15.5) |

Abbreviations: CI, confidence interval; IU, international units; GMT, geometric mean titer; GMFR, geometric mean fold-rise, comparing postbaseline to baseline titer values; IU, international units; LLOQ, lower limit of quantitation; RSV, respiratory syncytial virus; ULOQ, upper limit of quantitation.

95% CI was calculated based on the t-distribution of the log-transformed values for GMT, then back-transformed to the original scale for presentation; 95% CI for other measures were calculated using the Clopper-Pearson method.

For geometric mean fold rise (GMFR), comparing post-baseline to baseline titer values, antibody values reported as below LLOQ at baseline were replaced by LLOQ.

For GMT calculations, antibody values reported as below LLOQ were replaced by 0.5 × LLOQ.

RSV-A (IU/mL): LLOQ = 11, ULOQ = 176 050.

RSV-B (IU/mL): LLOQ = 8, ULOQ = 111 998.

^a^Number of participants in any per-protocol set.

^b^Number of participants with nonmissing baseline data.

^c^Number of participants with nonmissing data in the corresponding category at the corresponding time point.
