# Supplementary material for: Safety and Immunogenicity of an mRNA-Based RSV Vaccine Including a 12-Month Booster in a Phase 1 Clinical Trial in Healthy Older Adults
Source: J Infect Dis. 2024 Feb 22;230(3):e647–56. doi: 10.1093/infdis/jiae081 (PMC11420773; doi:10.1093/infdis/jiae081)
Supplement: jiae081_Supplementary_Data [file jiae081_supplementary_data.zip › Shaw_Supplementary_Table 11.docx]

**Table S11. Binding Antibody Concentrations After the Booster Injection (Per-protocol Booster Set)**

|  |  | **mRNA-1345/Placebo** | | | | | **mRNA-1345/mRNA-1345** | | | | |
| --- | --- | --- | --- | --- | --- | --- | --- | --- | --- | --- | --- |
|  | **Placebo/**  **Placebo** | **mRNA-1345**  **12.5 µg/**  **Placebo** | **mRNA-1345**  **25 µg/**  **Placebo** | **mRNA-1345**  **50 µg/**  **Placebo** | **mRNA-1345**  **100 µg/**  **Placebo** | **mRNA-1345**  **200 µg/**  **Placebo** | **mRNA-1345**  **12.5 µg/**  **mRNA-1345  12.5 µg** | **mRNA-1345**  **25 µg/**  **mRNA-1345  25 µg** | **mRNA-1345**  **50 µg/**  **mRNA-1345  50 µg** | **mRNA-1345**  **100 µg/**  **mRNA-1345 100 µg** | **mRNA-1345**  **200 µg/**  **mRNA-1345**  **200 µg** |
|  | **N= 51^a^** | **N = 20^a^** | **N = 19^a^** | **N = 21^a^** | **N = 17^a^** | **N = 17^a^** | **N = 21^a^** | **N = 22^a^** | **N = 18^a^** | **N = 17^a^** | **N = 20^a^** |
| **PreF-binding antibody (AU/mL)** | | | | | | |  |  |  |  |  |
| Baseline, n^b^ | 51 | 20 | 19 | 21 | 17 | 17 | 21 | 22 | 18 | 17 | 20 |
| GMC  (95% CI) | 8190.2  (6573.5, 10 204.6) | 7423.4  (5174.3, 10 650.1) | 10 201.7  (7547.1, 13 789.9) | 7436.4  (5566.2, 9935.1) | 7828.5  (5242.2, 11 690.8) | 9564.7  (6790.6, 13 472.1) | 8487.3  (6078.0, 11 851.7) | 7867.0  (5665.6, 10 923.8) | 6356.4  (4262.4, 9479.2) | 4764.4  (3097.6, 7327.9) | 9512.4  (6109.7, 14 810.1) |
| Month 1  (day 29), n^c^ | 49 | 19 | 19 | 19 | 17 | 17 | 21 | 21 | 17 | 17 | 20 |
| GMC  (95% CI) | 8603.2  (6658.7, 11 115.4) | 56 017.7  (37 508.3, 83 661.0) | 83 521.7  (583 15.4, 119 623.2) | 67 595.2  (45 726.3, 99 922.9) | 85 623.6  (62 325.1, 117 631.6) | 83 789.7  (54 227.0, 129 469.1) | 67 334.5  (46 102.6, 98 344.4) | 67 968.5  (51 064.3, 90 468.6) | 46 428.5  (34 218.1, 62 995.8) | 67 833.8  (52 305.9, 87971.4) | 141 704.0  (95 794.6, 20 9615.5) |
| GMFR  (95% CI) | 1.1  (0.9, 1.2) | 7.2  (4.9, 10.7) | 8.2  (6.4, 10.5) | 9.5  (6.9, 13.1) | 10.9  (7.3, 16.4) | 8.8  (5.5, 14.1) | 7.9  (5.5, 11.5) | 8.5  (5.7, 12.7) | 7.8  (5.1, 11.9) | 14.2  (9.7, 21.0) | 14.9  (9.4, 23.7) |
| Month 2  (day 57), n^c^ | 51 | 19 | 17 | 21 | 15 | 15 | 20 | 21 | 17 | 15 | 19 |
| GMC  (95% CI) | 8628.9  (6836.7, 10 891.0) | 33 628.0  (23 125.3, 48 900.7) | 61 883.3  (37 997.2, 100 785.0) | 57 715.5  (40 692.2, 81 860.5) | 64 787.8  (49 141.1, 85 416.3) | 82 741.8  (56 453.3, 121 272.0) | 51 419.0  (36 357.7, 72 719.5) | 48 484.1  (36 917.4, 63 674.8) | 38 085.1  (27 997.2, 51 807.7) | 47 687.4  (35 974.0, 63 214.7) | 95 881.1  (68 327.4,  13 4546.2) |
| GMFR  (95% CI) | 1.1  (0.9, 1.2) | 4.6  (3.4, 6.2) | 6.4  (4.4, 9.3) | 7.8  (5.8, 10.4) | 8.0  (5.3, 12.2) | 8.6  (5.6, 13.3) | 6.0  (4.1, 8.8) | 6.4  (4.6, 9.0) | 6.0  (4.0, 9.0) | 8.7  (6.1, 12.5) | 9.8  (6.2, 15.4) |
| Month 3 (day 85), n^c^ | 48 | 19 | 19 | 19 | 17 | 17 | 20 | 21 | 17 | 15 | 19 |
| GMC  (95% CI) | 8690.9  (6758.3, 11 176.0) | 29 505.4  (19 021.2, 45 768.4) | 53 837.7  (36 023.1, 80 462.0) | 44 485.5  (30 784.3, 64 284.6) | 48 126.6  (36 086.1, 64 184.6) | 67 924.8  (46 715.5, 98 763.3) | 42 543.7  (29 858.4, 60 618.2) | 40 991.5  (31 346.9, 53 603.5) | 30 140.7  (22 452.2, 40 462.1) | 40 080.7  (30 337.7, 52 952.6) | 73 689.5  (54 038.1, 100 487.4) |
| GMFR  (95% CI) | 1.0  (0.9, 1.2) | 4.1  (2.8, 5.9) | 5.3  (3.9, 7.1) | 5.9  (4.3, 8.1) | 6.2  (4.3, 8.8) | 7.1  (4.7, 10.6) | 5.0  (3.5, 7.1) | 5.4  (3.8, 7.7) | 4.7  (2.8, 8.0) | 8.3  (5.5, 12.4) | 7.5  (5.1, 11.1) |
| Month 6 (day 169), n^c^ | 50 | 20 | 19 | 20 | 16 | 17 | 21 | 20 | 16 | 16 | 18 |
| GMC  (95% CI) | 8680.6  (6901.9, 10 917.6) | 19 348.2  (12 463.7, 30 035.5) | 35 128.0  (24 920.6, 49 516.4) | 29 286.4  (20 634.2, 41 566.6) | 25 959.0  (16 676.9, 40 407.5) | 43 789.5  (30 145.4, 63 608.9) | 26 430.3  (18 961.3, 36 841.4) | 22 978.7  (16 841.2, 31 353.0) | 24 360.1  (17 444.2, 34 018.0) | 25 890.7  (18 513.5, 36 207.6) | 43 032.2  (30 244.3, 61 227.0) |
| GMFR  (95% CI) | 1.0  (0.9, 1.2) | 2.6  (1.9, 3.6) | 3.4  (2.7, 4.5) | 4.1  (3.2, 5.4) | 3.4  (2.1, 5.4) | 4.6  (3.1, 6.8) | 3.1  (2.3, 4.3) | 3.1  (2.2, 4.4) | 4.0  (2.7, 5.8) | 5.9  (4.2, 8.2) | 4.9  (3.5, 6.9) |
| Month 12  (day 365), n^a^ | 49 | 20 | 19 | 21 | 16 | 17 | 20 | 22 | 18 | 17 | 20 |
| GMC  (95% CI) | 9660.9  (7556.6, 12 351.2) | 14 258.9  (9505.3, 21 389.7) | 25 688.9  (17 812.8, 37 047.4) | 21 110.3  (14 157.0, 31 479.0) | 21 338.6  (15 334.9, 29 692.6) | 33 097.5  (21 894.2, 50 033.5) | 19 594.4  (13 801.3, 27 819.1) | 18 363.1  (14 106.8, 23 903.6) | 17 587.9  (12 839.2, 24 093.0) | 18 152.3  (13 493.9, 24 418.8) | 29 242.4  (20 988.1, 40 743.1) |
| GMFR  (95% CI) | 1.2  (1.0, 1.4) | 1.9  (1.5, 2.6) | 2.5  (1.9, 3.3) | 2.8  (2.1, 3.9) | 3.0  (2.2, 4.1) | 3.5  (2.4, 5.1) | 2.3  (1.7, 3.0) | 2.3  (1.7, 3.2) | 2.8  (2.1, 3.7) | 3.8  (2.5, 5.7) | 3.1  (2.3, 4.1) |
| Month 1 after booster  (day 393), n^c^ | 48 | 19 | 17 | 21 | 15 | 15 | 21 | 22 | 17 | 16 | 16 |
| GMC  (95% CI) | 10 377.5  (7744.3, 13 905.9) | 15 264.7  (10 006.8, 23 285.2) | 24 264.1  (16 052.9, 36 675.5) | 19 529.3  (13 247.8,  28 789.2) | 21 370.0  (15 429.4, 29 597.8) | 28 683.5  (17 632.7, 46 660.0) | 58 544.7  (44 442.8, 77 121.1) | 55 992.4  (41 343.3, 75 832.0) | 47 745.1  (37 949.0, 60 069.8) | 50 837.2 (40 245.7, 64 216.1) | 10 0271.5  (73 340.3,  13 7092.2) |
| GMFR  (95% CI) | 1.2  (1.0, 1.5) | 2.0  (1.5, 2.6) | 2.5  (1.9, 3.3) | 2.6  (2.0, 3.5) | 2.6  (1.8, 3.8) | 3.2  (2.1, 4.8) | 6.9  (5.0, 9.5) | 7.1  (4.7, 10.9) | 7.0  (4.8, 10.3) | 9.9  (6.7, 14.7) | 11.3  (6.7, 19.0) |
| Month 2 after booster  (day 421), n^c^ | 46 | 18 | 17 | 21 | 15 | 13 | 20 | 21 | 14 | 15 | 15 |
| GMC  (95% CI) | 10 016.7  (7411.2, 13 538.3) | 14 324.6  (8891.7, 23 076.8) | 23 002.9  (15 457.1, 34 232.5) | 20 365.4  (13 590.1, 30 518.3) | 21 506.8  (15 563.2, 29 720.3) | 22 625.4  (15 525.5, 32 972.2) | 49 039.5  (37 310.2, 64 456.3) | 42 736.6 (32 620.9, 55 989.2) | 41 995.4  (34 228.6, 51 524.6) | 43 498.8 (32 857.4, 57 586.5) | 80 742.2  (57 125.9,  114 121.6) |
| GMFR  (95% CI) | 1.2  (1.0, 1.5) | 1.9  (1.4, 2.5) | 2.4  (1.8, 3.1) | 2.7  (2.0, 3.7) | 2.6  (1.8, 4.0) | 2.6  (1.9, 3.7) | 6.0  (4.4, 8.2) | 5.5  (3.7, 8.2) | 6.5  (4.2, 9.9) | 8.4  (5.6, 12.6) | 7.5  (4.5, 12.6) |
| **PostF-binding antibody (AU/mL)** | | | | | | | | | | | |
| Baseline  (day 1), n^b^ | 51 | 20 | 19 | 21 | 17 | 17 | 21 | 22 | 18 | 17 | 20 |
| GMC  (95% CI) | 11 897.3  (9492.3, 14 911.7) | 9470.9  (5527.8, 16 226.7) | 18 178.7  (12 815.0, 25 787.3) | 11 795.8  (8093.2, 17 192.4) | 11 309.8  (7292.2, 17 540.9) | 11 436.0  (7236.1, 18 073.7) | 11 687.4  (7780.4, 17 556.3) | 11 204.8  (7356.2, 17 066.6) | 11 455.9  (6935.1, 18 923.5) | 8196.1  (5300.6, 12 673.3) | 9688.3  (6370.4, 14 734.2) |
| Month 1  (day 29), n^c^ | 49 | 19 | 19 | 19 | 17 | 17 | 21 | 21 | 17 | 17 | 20 |
| GMC  (95% CI) | 12 376.1  (9402.2, 16 290.6) | 48 175.6  (28 347.8, 81 871.8) | 106 330.7  (66 919.6, 168 952.2) | 80 988.5  (50 069.3, 131 001.4) | 95 646.5  (62 744.3, 145 802.0) | 76 097.7  (43 596.4, 132 828.9) | 50 607.1  (33 023.5, 77 553.2) | 63 198.4  (44 113.3, 90 540.4) | 55 428.6  (37 275.2, 82 422.8) | 75 484.2  (49 666.6,  114 722.3) | 113 505.3  (69 965.3,  184 140.8) |
| GMFR  (95% CI) | 1.1  (0.9, 1.2) | 4.6  (3.5, 6.1) | 5.9  (4.0, 8.6) | 7.1  (4.9, 10.3) | 8.5  (6.1, 11.8) | 6.7  (4.4, 10.0) | 4.3  (3.2, 5.9) | 5.5  (3.6, 8.3) | 5.1  (3.3, 7.7) | 9.2  (5.6, 15.3) | 11.7  (7.8, 17.6) |
| Month 2  (day 57), n^c^ | 51 | 19 | 17 | 21 | 15 | 15 | 20 | 21 | 17 | 15 | 19 |
| GMC  (95% CI) | 12 308.4  (9560.5, 15 846.1) | 33 950.8  (20 084.4, 57 390.6) | 80 269.5  (45 099.8, 142 865.2) | 68 901.2  (46 598.9, 101 877.4) | 65 601.2  (42 862.5, 100 403.0) | 84 971.5  (55 660.7, 129 717.3) | 41 355.9  (26 925.0, 63 521.1) | 43 817.8  (30 368.9, 63 222.4) | 40 539.1  (28 037.3, 58 615.5) | 44 317.1  (31 371.1, 62 605.6) | 77 694.7  (49 889.2,  12 0997.4) |
| GMFR  (95% CI) | 1.0  (0.9, 1.2) | 3.5  (2.7, 4.6) | 4.0  (2.3, 6.9) | 5.8  (4.1, 8.3) | 6.0  (4.2, 8.6) | 7.2  (5.2, 10.0) | 3.5  (2.5, 4.9) | 4.0  (2.8, 5.8) | 3.7  (2.6, 5.2) | 5.1  (3.5, 7.5) | 7.8  (5.3, 11.3) |
| Month 3 (day 85), n^c^ | 48 | 19 | 19 | 19 | 17 | 17 | 20 | 21 | 17 | 15 | 19 |
| GMC  (95% CI) | 12 676.8  (9738.2, 16 502.2) | 27 527.7  (16 087.5, 47 103.4) | 71 365.7  (43 326.4, 117 551.1) | 51 582.2  (33 087.9, 80 413.7) | 50 048.0  (32 911.8, 76 106.4) | 61 205.8  (37 765.4, 99 195.2) | 34 169.9  (21 795.2, 53 570.7) | 37 909.6  (26 310.6, 54 622.2) | 32 212.5  (20 542.1, 50 513.0) | 49 290.7  (33 820.2, 71 837.9) | 65 030.3  (41 269.0, 102 472.4) |
| GMFR  (95% CI) | 1.0  (0.9, 1.2) | 2.9  (2.3, 3.6) | 3.9  (2.7, 5.8) | 4.3  (3.0, 6.1) | 4.4  (3.4, 5.9) | 5.4  (3.7, 7.8) | 2.9  (2.1, 4.0) | 3.5  (2.4, 5.0) | 2.9  (1.6, 5.4) | 5.8  (3.6, 9.4) | 6.5  (4.7, 9.1) |
| Month 6 (day 169), n^c^ | 50 | 20 | 19 | 20 | 16 | 17 | 21 | 20 | 16 | 16 | 18 |
| GMC  (95% CI) | 12 537.6  (9885.4, 15 901.4) | 17 898.1  (10 574.7, 30 293.2) | 49 643.2  (3 2067.9, 76 851.0) | 35 755.4  (22 807.3, 56 054.6) | 29 101.2  (17 316.8, 48 905.2) | 43 923.3  (27 434.0, 70 323.8) | 22 948.5  (15 182.9, 34 686.0) | 25 216.5  (17 158.6, 37 058.5) | 32 346.6  (21 100.3, 49 587.0) | 28 645.4  (18 683.5, 43 918.9) | 37 336.9  (23 582.3, 59 114.0) |
| GMFR  (95% CI) | 1.0  (0.9, 1.2) | 1.9  (1.6, 2.3) | 2.7  (1.9, 3.9) | 3.1  (2.2, 4.2) | 2.5  (1.7, 3.7) | 3.8  (2.7, 5.5) | 2.0  (1.5, 2.6) | 2.2  (1.5, 3.3) | 2.8  (2.0, 3.9) | 3.6  (2.4, 5.5) | 3.8  (2.8, 5.1) |
| Month 12  (day 365), n^c^ | 49 | 20 | 19 | 21 | 16 | 17 | 20 | 22 | 18 | 17 | 20 |
| GMC  (95% CI) | 13 768.8  (10464.4, 18116.6) | 14 215.2  (8494.5, 23 788.5) | 38 056.9  (24 754.7, 58 507.4) | 26112.6  (16743.6, 40724.0) | 21 867.0  (14 375.1, 33 263.4) | 29 542.1  (17 797.0, 49 038.2) | 18 877.0  (11 854.4, 30 059.6) | 18 970.9  (13 694.9, 26 279.6) | 24 961.2  (16 810.9, 37 062.8) | 21 331.2  (14 521.5, 31 334.2) | 26 458.5  (17 331.8, 40 391.0) |
| GMFR  (95% CI) | 1.2  (1.0, 1.4) | 1.5  (1.3, 1.8) | 2.1  (1.5, 2.9) | 2.2  (1.7, 3.0) | 2.1  (1.7, 2.6) | 2.6  (1.9, 3.5) | 1.6  (1.3, 2.1) | 1.7  (1.2, 2.4) | 2.2  (1.6, 3.0) | 2.6  (1.7, 3.9) | 2.7  (2.1, 3.5) |
| Month 1 after booster  (day 393), n^c^ | 48 | 19 | 17 | 21 | 15 | 15 | 21 | 22 | 17 | 16 | 16 |
| GMC  (95% CI) | 15 266.5  (11 384.6, 20 472.0) | 14 337.8  (8288.7, 24 801.5) | 40 164.7  (26 091.9, 61 827.7) | 25 409.4  (16 351.7, 39 484.2) | 22 357.6  (15 027.0, 33 264.4) | 25 724.6  (15 544.8, 42 570.9) | 42 528.0  (26 926.1, 67 170.1) | 43 548.3  (31 457.0, 60 287.3) | 51 063.9  (35 773.9, 72 888.8) | 42 833.5  (29 023.1, 63 215.5) | 69 767.6  (50 382.0, 96 612.1) |
| GMFR  (95% CI) | 1.2  (1.0, 1.4) | 1.5  (1.2, 1.9) | 2.1  (1.5, 3.1) | 2.2  (1.6, 2.8) | 1.8  (1.4, 2.4) | 2.6  (1.8, 3.7) | 3.6  (2.6, 5.1) | 3.9  (2.6, 5.9) | 4.1  (2.8, 5.9 | 5.1  (3.4, 7.7) | 8.1  (5.7, 11.6) |
| Month 2 after booster  (day 421), n^c^ | 46 | 18 | 17 | 21 | 15 | 13 | 20 | 21 | 14 | 15 | 15 |
| GMC  (95% CI) | 14 053.6  (10 269.3, 19 232.4) | 12 763.5  (7381.4, 22 069.9) | 38 643.5  (25 236.7, 59 172.5) | 24 872.8  (16 033.1, 38 586.4) | 22 798.9  (15 282.0, 34 013.4) | 23 563.3  (13 818.6, 40 179.8) | 37 522.9  (23 373.3, 60 238.3) | 36 646.6  (26 825.8, 50 062.9) | 48 252.4  (32 612.5, 71 392.9) | 37 282.8  (24 144.0, 57 571.6) | 63 106.3  (43 237.9, 92 104.4) |
| GMFR  (95% CI) | 1.2  (1.0, 1.4) | 1.5  (1.2, 1.8) | 2.1  (1.4, 3.0) | 2.1  (1.6, 2.8) | 1.9  (1.4, 2.4) | 2.6  (1.8, 3.6) | 3.3  (2.3, 4.6) | 3.2  (2.1, 4.8) | 3.5  (2.4, 5.3) | 4.4  (2.9, 6.5) | 6.1  (4.2, 9.0) |

Abbreviations: AU, arbitrary units; CI, confidence interval; IU, international units; GMC, geometric mean concentration; GMFR, geometric mean fold-rise, comparing postbaseline to baseline titer values; LLOQ, lower limit of quantitation; RSV, respiratory syncytial virus; ULOQ, upper limit of quantitation.

95% CI is calculated based on the t-distribution of the log-transformed values GMC, then back-transformed to the original scale for presentation; 95% CI for other measures are calculated using the Clopper-Pearson method.

For geometric mean fold rise (GMFR), comparing post-baseline to baseline titer values, antibody values reported as below LLOQ at baseline were replaced by LLOQ.

For GMC calculations, antibody values reported as below LLOQ were replaced by 0.5 × LLOQ.

PreF Binding antibody: LLOQ = 19.00 (AU/mL), ULOQ = 1 111 100 (AU/mL).

PostF Binding antibody: LLOQ = 16.00 (AU/mL), ULOQ=1 111 100 (AU/mL).

^a^Number of participants in any per-protocol set.

^b^Number of participants with nonmissing baseline data.

^c^Number of participants with nonmissing data in the corresponding category at the corresponding time point.
