# Supplementary material for: Safety and Immunogenicity of an mRNA-Based RSV Vaccine Including a 12-Month Booster in a Phase 1 Clinical Trial in Healthy Older Adults
Source: J Infect Dis. 2024 Feb 22;230(3):e647–56. doi: 10.1093/infdis/jiae081 (PMC11420773; doi:10.1093/infdis/jiae081)
Supplement: jiae081_Supplementary_Data [file jiae081_supplementary_data.zip › Shaw_Supplementary_Table 2.docx]

**Table S2. Adverse Events of Special Interest**

| **Medical Concept** | **Additional Notes** |
| --- | --- |
| Thrombocytopenia | - Platelet counts < 150 × 10^9^ cells per liter - Including but not limited to immune thrombocytopenia, platelet production decreased, thrombocytopenia, thrombocytopenic purpura, thrombotic thrombocytopenic purpura, or HELLP (hemolysis, elevated liver enzymes, and low platelet count) syndrome |
| New onset of or worsening of neurologic diseases | Neurologic diseases include the following:   - Guillain-Barré syndrome - Acute disseminated encephalomyelitis - Idiopathic peripheral facial nerve palsy (Bell’s palsy) - Seizures, including but not limited to febrile seizures and/or generalized seizures/convulsions |
| Anaphylaxis | - Anaphylaxis as defined per the protocol |
| Myocarditis/Pericarditis | - Myocarditis - Pericarditis - Myopericarditis |
