# Supplementary material for: Safety and Immunogenicity of an mRNA-Based RSV Vaccine Including a 12-Month Booster in a Phase 1 Clinical Trial in Healthy Older Adults
Source: J Infect Dis. 2024 Feb 22;230(3):e647–56. doi: 10.1093/infdis/jiae081 (PMC11420773; doi:10.1093/infdis/jiae081)
Supplement: jiae081_Supplementary_Data [file jiae081_supplementary_data.zip › Shaw_Supplementary_Table 3.docx]

**Table S3. Baseline Demographics – Booster Injection (Randomized Set)**

|  | **Placebo/**  **Placebo**  **N = 52^a^** | **mRNA-1345**  **12.5 µg/**  **Placebo**  **N = 20^a^** | **mRNA-1345**  **12.5 µg/**  **mRNA-1345**  **12.5 µg**  **N = 21^a^** | **mRNA-1345**  **25 µg/**  **Placebo**  **N = 20^a^** | **mRNA-1345**  **25 µg/**  **mRNA-1345**  **25 µg**  **N = 22^a^** | **mRNA-1345 50 µg/**  **Placebo**  **N = 21^a^** | **mRNA-1345 50 µg/**  **mRNA-1345  50 µg**  **N = 18^a^** | **mRNA-1345 100 µg/**  **Placebo**  **N = 18^a^** | **mRNA-1345 100 µg/**  **mRNA-1345  100 µg**  **N = 18^a^** | **mRNA-1345 200 µg/**  **Placebo**  **N = 17^a^** | **mRNA-1345 200 µg/**  **mRNA-1345  200 µg**  **N = 20^a^** |
| --- | --- | --- | --- | --- | --- | --- | --- | --- | --- | --- | --- |
| **Mean (SD) age, y** | 70.0 (3.7) | 69.7 (3.6) | 70.0 (3.2) | 70.9 (3.6) | 70.7 (3.9) | 70.7 (4.0) | 70.1 (4.1) | 70.6 (4.0) | 70.4 (4.5) | 70.1 (4.7) | 69.7 (3.8) |
| **Sex, n^b^ (%)** |  |  |  |  |  |  |  |  |  |  |  |
| Female | 28 (53.8) | 13 (65.0) | 12 (57.1) | 12 (60.0) | 12 (54.5) | 9 (42.9) | 10 (55.6) | 9 (50.0) | 9 (50.0) | 11 (64.7) | 9 (45.0) |
| Male | 24 (46.2) | 7 (35.0) | 9 (42.9) | 8 (40.0) | 10 (45.5) | 12 (57.1) | 8 (44.4) | 9 (50.0) | 9 (50.0) | 6 (35.3) | 11 (55.0) |
| **Race, n^b^ (%)** |  |  |  |  |  |  |  |  |  |  |  |
| White | 44 (84.6) | 18 (90.0) | 21 (100.0) | 18 (90.0) | 21 (95.5) | 18 (85.7) | 18 (100.0) | 15 (83.3) | 17 (94.4) | 17 (100.0) | 18 (90.0) |
| Black or African American | 5 (9.6) | 2 (10.0) | 0 | 1 (5.0) | 0 | 1 (4.8) | 0 | 3 (16.7) | 1 (5.6) | 0 | 1 (5.0) |
| Asian | 1 (1.9) | 0 | 0 | 1 (5.0) | 0 | 1 (4.8) | 0 | 0 | 0 | 0 | 0 |
| American Indian or Alaska Native | 0 | 0 | 0 | 0 | 0 | 0 | 0 | 0 | 0 | 0 | 1 (5.0) |
| Native Hawaiian or Other Pacific Islander | 1 (1.9) | 0 | 0 | 0 | 0 | 0 | 0 | 0 | 0 | 0 | 0 |
| Multiple | 0 | 0 | 0 | 0 | 0 | 1 (4.8) | 0 | 0 | 0 | 0 | 0 |
| Other | 1 (1.9) | 0 | 0 | 0 | 1 (4.5) | 0 | 0 | 0 | 0 | 0 | 0 |
| **Ethnicity, n^b^ (%)** |  |  |  |  |  |  |  |  |  |  |  |
| Hispanic or Latino | 4 (7.7) | 0 | 2 (9.5) | 2 (10.0) | 0 | 1 (4.8) | 1 (5.6) | 0 | 0 | 1 (5.9) | 2 (10.0) |
| Not Hispanic or Latino | 48 (92.3) | 18 (90.0) | 19(90.5) | 18 (90.0) | 22 (100.0) | 20 (95.2) | 17 (94.4) | 18 (100.0) | 18 (100.0) | 16 (94.1) | 18 (90.0) |
| Not reported | 0 | 2 (10.0) | 0 | 0 | 0 | 0 | 0 | 0 | 0 | 0 | 0 |
| **Mean (SD) BMI , kg/m^2^** | 28.2 (4.0) | 28.0 (3.8) | 29.3 (3.3) | 27.3 (3.5) | 25.8 (4.4) | 27.8 (3.5) | 28.4 (3.6) | 27.9 (3.5) | 27.7 (3.6) | 25.7 (4.4) | 28.1 (3.4) |

Abbreviation: BMI, body mass index.

^a^Number of randomly assigned participants.

^b^Number of randomly assigned participants in the category with nonmissing data.
