# Supplementary material for: Safety and Immunogenicity of an mRNA-Based RSV Vaccine Including a 12-Month Booster in a Phase 1 Clinical Trial in Healthy Older Adults
Source: J Infect Dis. 2024 Feb 22;230(3):e647–56. doi: 10.1093/infdis/jiae081 (PMC11420773; doi:10.1093/infdis/jiae081)
Supplement: jiae081_Supplementary_Data [file jiae081_supplementary_data.zip › Shaw_Supplementary_Table 4.docx]

**Table S4.** **Summary of Solicited Adverse Reactions Through 7 Days by Toxicity Grade for Each Vaccination After the First Injection (Solicited Safety Set)**

|  | **Placebo** | **mRNA-1345** | | | | | |
| --- | --- | --- | --- | --- | --- | --- | --- |
|  | **N = 58^a^** | **12.5 µg**  **N = 46^a^** | **25 µg**  **N = 48^a^** | **50 µg**  **N = 47^a^** | **100 µg**  **N = 48^a^** | **200 µg**  **N = 47^a^** | **Total**  **N = 236^a^** |
| **n (%)^b^** |  |  |  |  |  |  |  |
| Solicited adverse reactions^c^ | 55 | 46 | 44 | 47 | 47 | 47 | 231 |
| Any solicited adverse reactions | 25 (45.5) | 31 (67.4) | 35 (79.5) | 34 (72.3) | 42 (89.4) | 42 (89.4) | 184 (79.7) |
| 95% confidence interval | (32.0, 59.4) | (52.0, 80.5) | (64.7, 90.2) | (57.4, 84.4) | (76.9, 96.5) | (76.9, 96.5) | (73.9, 84.7) |
| Grade 1 | 14 (25.5) | 23 (50.0) | 27 (61.4) | 25 (53.2) | 27 (57.4) | 17 (36.2) | 119 (51.5) |
| Grade 2 | 8 (14.5) | 6 (13.0) | 5 (11.4) | 4 (8.5) | 11 (23.4) | 18 (38.3) | 44 (19.0) |
| Grade 3 | 3 (5.5) | 2 (4.3) | 3 (6.8) | 5 (10.6) | 4 (8.5) | 7 (14.9) | 21 (9.1) |
| Solicited local ARs^c^ | 55 | 46 | 44 | 47 | 47 | 47 | 231 |
| Any solicited local ARs | 7 (12.7) | 23 (50.0) | 29 (65.9) | 29 (61.7) | 35 (74.5) | 37 (78.7) | 153 (66.2) |
| 95% confidence interval | (5.3, 24.5) | (34.9, 65.1) | (50.1, 79.5) | (46.4, 75.5) | (59.7, 86.1) | (64.3, 89.3) | (59.7, 72.3) |
| Grade 1 | 4 (7.3) | 23 (50.0) | 24 (54.5) | 28 (59.6) | 31 (66.0) | 28 (59.6) | 134 (58.0) |
| Grade 2 | 0 | 0 | 2 (4.5) | 1 (2.1) | 4 (8.5) | 8 (17.0) | 15 (6.5) |
| Grade 3 | 3 (5.5) | 0 | 3 (6.8) | 0 | 0 | 1 (2.1) | 4 (1.7) |
| Injection site pain | 55 | 46 | 44 | 47 | 47 | 47 | 231 |
| Any | 7 (12.7) | 23 (50.0) | 29 (65.9) | 29 (61.7) | 35 (74.5) | 37 (78.7) | 153 (66.2) |
| Grade 1 | 4 (7.3) | 23 (50.0) | 24 (54.5) | 28 (59.6) | 32 (68.1) | 28 (59.6) | 135 (58.4) |
| Grade 2 | 0 | 0 | 2 (4.5) | 1 (2.1) | 3 (6.4) | 8 (17.0) | 14 (6.1) |
| Grade 3 | 3 (5.5) | 0 | 3 (6.8) | 0 | 0 | 1 (2.1) | 4 (1.7) |
| Erythema | 55 | 46 | 44 | 47 | 47 | 47 | 231 |
| Any | 0 | 2 (4.3) | 1 (2.3) | 0 | 2 (4.3) | 1 (2.1) | 6 (2.6) |
| Grade 1 | 0 | 2 (4.3) | 1 (2.3) | 0 | 1 (2.1) | 1 (2.1) | 5 (2.2) |
| Grade 2 | 0 | 0 | 0 | 0 | 1 (2.1) | 0 | 1 (0.4) |
| Grade 3 | 0 | 0 | 0 | 0 | 0 | 0 | 0 |
| Swelling | 55 | 46 | 44 | 47 | 47 | 47 | 231 |
| Any | 0 | 0 | 0 | 1 (2.1) | 2 (4.3) | 2 (4.3) | 5 (2.2) |
| Grade 1 | 0 | 0 | 0 | 1 (2.1) | 1 (2.1) | 2 (4.3) | 4 (1.7) |
| Grade 2 | 0 | 0 | 0 | 0 | 1 (2.1) | 0 | 1 (0.4) |
| Grade 3 | 0 | 0 | 0 | 0 | 0 | 0 | 0 |
| Solicited systemic ARs^c^ | 55 | 46 | 44 | 47 | 47 | 47 | 231 |
| Any solicited systemic ARs | 25 (45.5) | 23 (50.0) | 23 (52.3) | 25 (53.2) | 37 (78.7) | 31 (66.0) | 139 (60.2) |
| 95% confidence interval | (32.0, 59.4) | (34.9, 65.1) | (36.7, 67.5) | (38.1, 67.9) | (64.3, 89.3) | (50.7, 79.1) | (53.5, 66.5) |
| Grade 1 | 16 (29.1) | 15 (32.6) | 16 (36.4) | 16 (34.0) | 24 (51.1) | 6 (12.8) | 77 (33.3) |
| Grade 2 | 8 (14.5) | 6 (13.0) | 6 (13.6) | 4 (8.5) | 9 (19.1) | 18 (38.3) | 43 (18.6) |
| Grade 3 | 1 (1.8) | 2 (4.3) | 1 (2.3) | 5 (10.6) | 4 (8.5) | 7 (14.9) | 19 (8.2) |
| Fever | 54 | 46 | 44 | 47 | 47 | 47 | 231 |
| Any | 1 (1.9) | 1 (2.2) | 2 (4.5) | 1 (2.1) | 6 (12.8) | 2 (4.3) | 12 (5.2) |
| Grade 1 | 1 (1.9) | 1 (2.2) | 2 (4.5) | 0 | 5 (10.6) | 2 (4.3) | 10 (4.3) |
| Grade 2 | 0 | 0 | 0 | 1 (2.1) | 1 (2.1) | 0 | 2 (0.9) |
| Grade 3 | 0 | 0 | 0 | 0 | 0 | 0 | 0 |
| Headache | 55 | 46 | 44 | 47 | 47 | 47 | 231 |
| Any | 8 (14.5) | 13 (28.3) | 16 (36.4) | 15 (31.9) | 21 (44.7) | 19 (40.4) | 84 (36.4) |
| Grade 1 | 5 (9.1) | 11 (23.9) | 14 (31.8) | 9 (19.1) | 16 (34.0) | 10 (21.3) | 60 (26.0) |
| Grade 2 | 2 (3.6) | 0 | 1 (2.3) | 3 (6.4) | 2 (4.3) | 8 (17.0) | 14 (6.1) |
| Grade 3 | 1 (1.8) | 2 (4.3) | 1 (2.3) | 3 (6.4) | 3 (6.4) | 1 (2.1) | 10 (4.3) |
| Fatigue | 55 | 46 | 44 | 47 | 47 | 47 | 231 |
| Any | 20 (36.4) | 11 (23.9) | 15 (34.1) | 14 (29.8) | 28 (59.6) | 27 (57.4) | 95 (41.1) |
| Grade 1 | 12 (21.8) | 5 (10.9) | 10 (22.7) | 9 (19.1) | 17 (36.2) | 10 (21.3) | 51 (22.1) |
| Grade 2 | 8 (14.5) | 6 (13.0) | 5 (11.4) | 2 (4.3) | 9 (19.1) | 11 (23.4) | 33 (14.3) |
| Grade 3 | 0 | 0 | 0 | 3 (6.4) | 2 (4.3) | 6 (12.8) | 11 (4.8) |
| Myalgia | 55 | 46 | 44 | 47 | 47 | 47 | 231 |
| Any | 10 (18.2) | 9 (19.6) | 11 (25.0) | 13 (27.7) | 17 (36.2) | 23 (48.9) | 73 (31.6) |
| Grade 1 | 6 (10.9) | 6 (13.0) | 8 (18.2) | 7 (14.9) | 9 (19.1) | 11 (23.4) | 41 (17.7) |
| Grade 2 | 4 (7.3) | 3 (6.5) | 3 (6.8) | 4 (8.5) | 8 (17.0) | 8 (17.0) | 26 (11.3) |
| Grade 3 | 0 | 0 | 0 | 2 (4.3) | 0 | 4 (8.5) | 6 (2.6) |
| Arthralgia | 55 | 46 | 44 | 47 | 47 | 47 | 231 |
| Any | 13 (23.6) | 6 (13.0) | 6 (13.6) | 13 (27.7) | 16 (34.0) | 16 (34.0) | 57 (24.7) |
| Grade 1 | 10 (18.2) | 4 (8.7) | 4 (9.1) | 9 (19.1) | 11 (23.4) | 11 (23.4) | 39 (16.9) |
| Grade 2 | 3 (5.5) | 2 (4.3) | 2 (4.5) | 2 (4.3) | 5 (10.6) | 2 (4.3) | 13 (5.6) |
| Grade 3 | 0 | 0 | 0 | 2 (4.3) | 0 | 3 (6.4) | 5 (2.2) |
| Nausea/vomiting | 55 | 46 | 44 | 47 | 47 | 47 | 231 |
| Any | 5 (9.1) | 2 (4.3) | 3 (6.8) | 3 (6.4) | 7 (14.9) | 5 (10.6) | 20 (8.7) |
| Grade 1 | 4 (7.3) | 1 (2.2) | 3 (6.8) | 2 (4.3) | 5 (10.6) | 3 (6.4) | 14 (6.1) |
| Grade 2 | 1 (1.8) | 1 (2.2) | 0 | 0 | 2 (4.3) | 2 (4.3) | 5 (2.2) |
| Grade 3 | 0 | 0 | 0 | 1 (2.1) | 0 | 0 | 1 (0.4) |
| Lymphadenopathy | 55 | 46 | 44 | 47 | 47 | 47 | 231 |
| Any | 3 (5.5) | 3 (6.5) | 5 (11.4) | 3 (6.4) | 7 (14.9) | 4 (8.5) | 22 (9.5) |
| Grade 1 | 3 (5.5) | 3 (6.5) | 4 (9.1) | 3 (6.4) | 7 (14.9) | 3 (6.4) | 20 (8.7) |
| Grade 2 | 0 | 0 | 1 (2.3) | 0 | 0 | 1 (2.1) | 2 (0.9) |
| Grade 3 | 0 | 0 | 0 | 0 | 0 | 0 | 0 |
| Chills | 55 | 46 | 44 | 47 | 47 | 47 | 231 |
| Any | 3 (5.5) | 3 (6.5) | 2 (4.5) | 4 (8.5) | 9 (19.1) | 17 (36.2) | 35 (15.2) |
| Grade 1 | 1 (1.8) | 3 (6.5) | 1 (2.3) | 1 (2.1) | 5 (10.6) | 8 (17.0) | 18 (7.8) |
| Grade 2 | 2 (3.6) | 0 | 1 (2.3) | 2 (4.3) | 4 (8.5) | 9 (19.1) | 16 (6.9) |
| Grade 3 | 0 | 0 | 0 | 1 (2.1) | 0 | 0 | 1 (0.4) |

Abbreviation: AR, adverse reaction.

Participants were counted once based on the highest grade reported for each solicited symptom. The table does not include rows for grade 4 solicited ARs because no grade 4 solicited ARs were reported.

^a^Number of participants in the solicited safety set.

^b^Number (%) of participants in each group reporting the event.

^c^Number of participants in the solicited safety set who submitted any data for the event.
