# Supplementary material for: Safety and Immunogenicity of an mRNA-Based RSV Vaccine Including a 12-Month Booster in a Phase 1 Clinical Trial in Healthy Older Adults
Source: J Infect Dis. 2024 Feb 22;230(3):e647–56. doi: 10.1093/infdis/jiae081 (PMC11420773; doi:10.1093/infdis/jiae081)
Supplement: jiae081_Supplementary_Data [file jiae081_supplementary_data.zip › Shaw_Supplementary_Table 5.docx]

**Table S5. Summary of Solicited Adverse Reactions Through 7 Days by Toxicity Grade for Each Vaccination After the Booster Injection (Solicited Safety Set)**

|  |  | **mRNA-1345/Placebo** | | | | | | **mRNA-1345/mRNA-1345** | | | | | |
| --- | --- | --- | --- | --- | --- | --- | --- | --- | --- | --- | --- | --- | --- |
|  | **Placebo/**  **Placebo** | **mRNA-1345**  **12.5 µg/**  **Placebo** | **mRNA-1345**  **25 µg/**  **Placebo** | **mRNA-1345**  **50 µg/**  **Placebo** | **mRNA-1345**  **100 µg/**  **Placebo** | **mRNA-1345**  **200 µg/**  **Placebo** | **mRNA-1345/**  **Placebo Total** | **mRNA-1345**  **12.5 µg/**  **mRNA-1345 12.5 µg** | **mRNA-1345**  **25 µg/**  **mRNA-1345 25 µg** | **mRNA-1345**  **50 µg/**  **mRNA-1345 50 µg** | **mRNA-1345**  **100 µg/**  **mRNA-1345 100 µg** | **mRNA-1345**  **200 µg/**  **mRNA-1345**  **200 µg** | **mRNA-1345**  **/mRNA-1345**  **Total** |
|  | **N = 51^a^** | **N = 19^a^** | **N = 20^a^** | **N = 21^a^** | **N = 18^a^** | **N = 16^a^** | **N = 94^a^** | **N = 21^a^** | **N = 22^a^** | **N = 18^a^** | **N = 18^a^** | **N = 20^a^** | **N = 99^a^** |
| **After first Injection, n (%)^b^** | | | | | | |  |  |  |  |  |  |  |
| Solicited local AR, N^c^ | 48 | 19 | 19 | 21 | 17 | 16 | 92 | 21 | 19 | 18 | 18 | 20 | 96 |
| Any solicited local AR | 7 (14.6) | 8 (42.1) | 12 (63.2) | 10 (47.6) | 10 (58.8) | 12 (75.0) | 52 (56.5) | 12 (57.1) | 13 (68.4) | 13 (72.2) | 15 (83.3) | 16 (80.0) | 69 (71.9) |
| Grade 1 | 4 (8.3) | 8 (42.1) | 9 (47.4) | 10 (47.6) | 9 (52.9) | 11 (68.8) | 47 (51.1) | 12 (57.1) | 12 (63.2) | 13 (72.2) | 13 (72.2) | 12 (60.0) | 62 (64.6) |
| Grade 2 | 0 | 0 | 1 (5.3) | 0 | 1 (5.9) | 1 (6.3) | 3 (3.3) | 0 | 1 (5.3) | 0 | 2 (11.1) | 4 (20.0) | 7 (7.3) |
| Grade 3 | 3 (6.3) | 0 | 2 (10.5) | 0 | 0 | 0 | 2 (2.2) | 0 | 0 | 0 | 0 | 0 | 0 |
| Injection site pain | 48 | 19 | 19 | 21 | 17 | 16 | 92 | 21 | 19 | 18 | 18 | 20 | 96 |
| Any | 7 (14.6) | 8 (42.1) | 12 (63.2) | 10 (47.6) | 10 (58.8) | 12 (75.0) | 52 (56.5) | 12 (57.1) | 13 (68.4) | 13 (72.2) | 15 (83.3) | 16 (80.0) | 69 (71.9) |
| Grade 1 | 4 (8.3) | 8 (42.1) | 9 (47.4) | 10 (47.6) | 9 (52.9) | 11 (68.8) | 47 (51.1) | 12 (57.1) | 12 (63.2) | 13 (72.2) | 13 (72.2) | 12 (60.0) | 62 (64.6) |
| Grade 2 | 0 | 0 | 1 (5.3) | 0 | 1 (5.9) | 1 (6.3) | 3 (3.3) | 0 | 1 (5.3) | 0 | 2 (11.1) | 4 (20.0) | 7 (7.3) |
| Grade 3 | 3 (6.3) | 0 | 2 (10.5) | 0 | 0 | 0 | 2 (2.2) | 0 | 0 | 0 | 0 | 0 | 0 |
| Erythema | 48 | 19 | 19 | 21 | 17 | 16 | 92 | 21 | 19 | 18 | 18 | 20 | 96 |
| Any | 0 | 2 (10.5) | 0 | 0 | 0 | 1 (6.3) | 3 (3.3) | 0 | 1 (5.3) | 0 | 1 (5.6) | 0 | 2 (2.1) |
| Grade 1 | 0 | 2 (10.5) | 0 | 0 | 0 | 1 (6.3) | 3 (3.3) | 0 | 1 (5.3) | 0 | 1 (5.6) | 0 | 2 (2.1) |
| Grade 2 | 0 | 0 | 0 | 0 | 0 | 0 | 0 | 0 | 0 | 0 | 0 | 0 | 0 |
| Grade 3 | 0 | 0 | 0 | 0 | 0 | 0 | 0 | 0 | 0 | 0 | 0 | 0 | 0 |
| Swelling | 48 | 19 | 19 | 21 | 17 | 16 | 92 | 21 | 19 | 18 | 18 | 20 | 96 |
| Any | 0 | 0 | 0 | 1 (4.8) | 0 | 1 (6.3) | 2 (2.2) | 0 | 0 | 0 | 1 (5.6) | 1 (5.0) | 2 (2.1) |
| Grade 1 | 0 | 0 | 0 | 1 (4.8) | 0 | 1 (6.3) | 2 (2.2) | 0 | 0 | 0 | 1 (5.6) | 1 (5.0) | 2 (2.1) |
| Grade 2 | 0 | 0 | 0 | 0 | 0 | 0 | 0 | 0 | 0 | 0 | 0 | 0 | 0 |
| Grade 3 | 0 | 0 | 0 | 0 | 0 | 0 | 0 | 0 | 0 | 0 | 0 | 0 | 0 |
| Solicited systemic AR, N^c^ | 48 | 19 | 19 | 21 | 17 | 16 | 92 | 21 | 19 | 18 | 18 | 20 | 96 |
| Any solicited systemic AR | 21 (43.8) | 8 (42.1) | 12 (63.2) | 10 (47.6) | 13 (76.5) | 10 (62.5) | 53 (57.6) | 9 (42.9) | 8 (42.1) | 10 (55.6) | 14 (77.8) | 12 (60.0) | 53 (55.2) |
| Grade 1 | 13 (27.1) | 4 (21.1) | 9 (47.4) | 8 (38.1) | 10 (58.8) | 3 (18.8) | 34 (37.0) | 7 (33.3) | 5 (26.3) | 7 (38.9) | 8 (44.4) | 2 (10.0) | 29 (30.2) |
| Grade 2 | 7 (14.6) | 2 (10.5) | 2 (10.5) | 1 (4.8) | 3 (17.6) | 5 (31.3) | 13 (14.1) | 2 (9.5) | 3 (15.8) | 1 (5.6) | 4 (22.2) | 7 (35.0) | 17 (17.7) |
| Grade 3 | 1 (2.1) | 2 (10.5) | 1 (5.3) | 1 (4.8) | 0 | 2 (12.5) | 6 (6.5) | 0 | 0 | 2 (11.1) | 2 (11.1) | 3 (15.0) | 7 (7.3) |
| Fever | 48 | 19 | 19 | 21 | 17 | 16 | 92 | 21 | 19 | 18 | 18 | 20 | 96 |
| Any | 0 | 0 | 2 (10.5) | 0 | 2 (11.8) | 0 | 4 (4.3) | 0 | 0 | 0 | 2 (11.1) | 1 (5.0) | 3 (3.1) |
| Grade 1 | 0 | 0 | 2 (10.5) | 0 | 2 (11.8) | 0 | 4 (4.3) | 0 | 0 | 0 | 2 (11.1) | 1 (5.0) | 3 (3.1) |
| Grade 2 | 0 | 0 | 0 | 0 | 0 | 0 | 0 | 0 | 0 | 0 | 0 | 0 | 0 |
| Grade 3 | 0 | 0 | 0 | 0 | 0 | 0 | 0 | 0 | 0 | 0 | 0 | 0 | 0 |
| Headache | 48 | 19 | 19 | 21 | 17 | 16 | 92 | 21 | 19 | 18 | 18 | 20 | 96 |
| Any | 7 (14.6) | 3 (15.8) | 8 (42.1) | 4 (19.0) | 4 (23.5) | 6 (37.5) | 25 (27.2) | 6 (28.6) | 6 (31.6) | 7 (38.9) | 12 (66.7) | 6 (30.0) | 37 (38.5) |
| Grade 1 | 5 (10.4) | 1 (5.3) | 6 (31.6) | 3 (14.3) | 3 (17.6) | 3 (18.8) | 16 (17.4) | 6 (28.6) | 6 (31.6) | 5 (27.8) | 10 (55.6) | 2 (10.0) | 29 (30.2) |
| Grade 2 | 1 (2.1) | 0 | 1 (5.3) | 0 | 1 (5.9) | 3 (18.8) | 5 (5.4) | 0 | 0 | 0 | 1 (5.6) | 4 (20.0) | 5 (5.2) |
| Grade 3 | 1 (2.1) | 2 (10.5) | 1 (5.3) | 1 (4.8) | 0 | 0 | 4 (4.3) | 0 | 0 | 2 (11.1) | 1 (5.6) | 0 | 3 (3.1) |
| Fatigue | 48 | 19 | 19 | 21 | 17 | 16 | 92 | 21 | 19 | 18 | 18 | 20 | 96 |
| Any | 17 (35.4) | 3 (15.8) | 7 (36.8) | 5 (23.8) | 8 (47.1) | 9 (56.3) | 32 (34.8) | 5 (23.8) | 7 (36.8) | 5 (27.8) | 13 (72.2) | 9 (45.0) | 39 (40.6) |
| Grade 1 | 10 (20.8) | 0 | 5 (26.3) | 3 (14.3) | 5 (29.4) | 5 (31.3) | 18 (19.6) | 4 (19.0) | 4 (21.1) | 4 (22.2) | 7 (38.9) | 3 (15.0) | 22 (22.9) |
| Grade 2 | 7 (14.6) | 3 (15.8) | 2 (10.5) | 1 (4.8) | 3 (17.6) | 2 (12.5) | 11 (12.0) | 1 (4.8) | 3 (15.8) | 1 (5.6) | 4 (22.2) | 4 (20.0) | 13 (13.5) |
| Grade 3 | 0 | 0 | 0 | 1 (4.8) | 0 | 2 (12.5) | 3 (3.3) | 0 | 0 | 0 | 2 (11.1) | 2 (10.0) | 4 (4.2) |
| Myalgia | 48 | 19 | 19 | 21 | 17 | 16 | 92 | 21 | 19 | 18 | 18 | 20 | 96 |
| Any | 7 (14.6) | 2 (10.5) | 6 (31.6) | 5 (23.8) | 7 (41.2) | 7 (43.8) | 27 (29.3) | 4 (19.0) | 4 (21.1) | 5 (27.8) | 8 (44.4) | 10 (50.0) | 31 (32.3) |
| Grade 1 | 4 (8.3) | 1 (5.3) | 4 (21.1) | 3 (14.3) | 4 (23.5) | 5 (31.3) | 17 (18.5) | 2 (9.5) | 4 (21.1) | 3 (16.7) | 3 (16.7) | 5 (25.0) | 17 (17.7) |
| Grade 2 | 3 (6.3) | 1 (5.3) | 2 (10.5) | 1 (4.8) | 3 (17.6) | 1 (6.3) | 8 (8.7) | 2 (9.5) | 0 | 2 (11.1) | 5 (27.8) | 3 (15.0) | 12 (12.5) |
| Grade 3 | 0 | 0 | 0 | 1 (4.8) | 0 | 1 (6.3) | 2 (2.2) | 0 | 0 | 0 | 0 | 2 (10.0) | 2 (2.1) |
| Arthralgia | 48 | 19 | 19 | 21 | 17 | 16 | 92 | 21 | 19 | 18 | 18 | 20 | 96 |
| Any | 10 (20.8) | 1 (5.3) | 4 (21.1) | 6 (28.6) | 7 (41.2) | 6 (37.5) | 24 (26.1) | 2 (9.5) | 1 (5.3) | 4 (22.2) | 6 (33.3) | 5 (25.0) | 18 (18.8) |
| Grade 1 | 8 (16.7) | 0 | 2 (10.5) | 4 (19.0) | 5 (29.4) | 5 (31.3) | 16 (17.4) | 1 (4.8) | 1 (5.3) | 3 (16.7) | 3 (16.7) | 3 (15.0) | 11 (11.5) |
| Grade 2 | 2 (4.2) | 1 (5.3) | 2 (10.5) | 1 (4.8) | 2 (11.8) | 0 | 6 (6.5) | 1 (4.8) | 0 | 1 (5.6) | 3 (16.7) | 1 (5.0) | 6 (6.3) |
| Grade 3 | 0 | 0 | 0 | 1 (4.8) | 0 | 1 (6.3) | 2 (2.2) | 0 | 0 | 0 | 0 | 1 (5.0) | 1 (1.0) |
| Nausea/vomiting | 48 | 19 | 19 | 21 | 17 | 16 | 92 | 21 | 19 | 18 | 18 | 20 | 96 |
| Any | 3 (6.3) | 1 (5.3) | 2 (10.5) | 1 (4.8) | 0 | 2 (12.5) | 6 (6.5) | 1 (4.8) | 1 (5.3) | 2 (11.1) | 5 (27.8) | 0 | 9 (9.4) |
| Grade 1 | 3 (6.3) | 0 | 2 (10.5) | 0 | 0 | 2 (12.5) | 4 (4.3) | 1 (4.8) | 1 (5.3) | 2 (11.1) | 4 (22.2) | 0 | 8 (8.3) |
| Grade 2 | 0 | 1 (5.3) | 0 | 0 | 0 | 0 | 1 (1.1) | 0 | 0 | 0 | 1 (5.6) | 0 | 1 (1.0) |
| Grade 3 | 0 | 0 | 0 | 1 (4.8) | 0 | 0 | 1 (1.1) | 0 | 0 | 0 | 0 | 0 | 0 |
| Lymphadenopathy | 48 | 19 | 19 | 21 | 17 | 16 | 92 | 21 | 19 | 18 | 18 | 20 | 96 |
| Any | 2 (4.2) | 2 (10.5) | 4 (21.1) | 3 (14.3) | 2 (11.8) | 2 (12.5) | 13 (14.1) | 1 (4.8) | 1 (5.3) | 0 | 3 (16.7) | 1 (5.0) | 6 (6.3) |
| Grade 1 | 2 (4.2) | 2 (10.5) | 3 (15.8) | 3 (14.3) | 2 (11.8) | 1 (6.3) | 11 (12.0) | 1 (4.8) | 1 (5.3) | 0 | 3 (16.7) | 1 (5.0) | 6 (6.3) |
| Grade 2 | 0 | 0 | 1 (5.3) | 0 | 0 | 1 (6.3) | 2 (2.2) | 0 | 0 | 0 | 0 | 0 | 0 |
| Grade 3 | 0 | 0 | 0 | 0 | 0 | 0 | 0 | 0 | 0 | 0 | 0 | 0 | 0 |
| Chills | 48 | 19 | 19 | 21 | 17 | 16 | 92 | 21 | 19 | 18 | 18 | 20 | 96 |
| Any | 2 (4.2) | 0 | 2 (10.5) | 2 (9.5) | 3 (17.6) | 5 (31.3) | 12 (13.0) | 1 (4.8) | 0 | 0 | 5 (27.8) | 6 (30.0) | 12 (12.5) |
| Grade 1 | 1 (2.1) | 0 | 1 (5.3) | 1 (4.8) | 2 (11.8) | 4 (25.0) | 8 (8.7) | 1 (4.8) | 0 | 0 | 3 (16.7) | 2 (10.0) | 6 (6.3) |
| Grade 2 | 1 (2.1) | 0 | 1 (5.3) | 0 | 1 (5.9) | 1 (6.3) | 3 (3.3) | 0 | 0 | 0 | 2 (11.1) | 4 (20.0) | 6 (6.3) |
| Grade 3 | 0 | 0 | 0 | 1 (4.8) | 0 | 0 | 1 (1.1) | 0 | 0 | 0 | 0 | 0 | 0 |
| **After booster Injection, n (%)** |  |  |  |  |  |  |  |  |  |  |  |  |  |
| Solicited local AR, N^c^ | 51 | 19 | 20 | 21 | 18 | 16 | 94 | 21 | 22 | 18 | 18 | 18 | 97 |
| Any solicited local AR | 5 (9.8) | 1 (5.3) | 1 (5.0) | 2 (9.5) | 3 (16.7) | 1 (6.3) | 8 (8.5) | 15 (71.4) | 15 (68.2) | 14 (77.8) | 14 (77.8) | 16 (88.9) | 74 (76.3) |
| Grade 1 | 4 (7.8) | 1 (5.3) | 1 (5.0) | 2 (9.5) | 2 (11.1) | 1 (6.3) | 7 (7.4) | 14 (66.7) | 12 (54.5) | 12 (66.7) | 11 (61.1) | 9 (50.0) | 58 (59.8) |
| Grade 2 | 0 | 0 | 0 | 0 | 0 | 0 | 0 | 0 | 2 (9.1) | 1 (5.6) | 2 (11.1) | 5 (27.8) | 10 (10.3) |
| Grade 3 | 1 (2.0) | 0 | 0 | 0 | 1 (5.6) | 0 | 1 (1.1) | 1 (4.8) | 1 (4.5) | 1 (5.6) | 1 (5.6) | 2 (11.1) | 6 (6.2) |
| Injection site pain | 51 | 19 | 20 | 21 | 18 | 16 | 94 | 21 | 22 | 18 | 18 | 18 | 97 |
| Any | 5 (9.8) | 1 (5.3) | 1 (5.0) | 2 (9.5) | 3 (16.7) | 1 (6.3) | 8 (8.5) | 15 (71.4) | 15 (68.2) | 14 (77.8) | 14 (77.8) | 16 (88.9) | 74 (76.3) |
| Grade 1 | 4 (7.8) | 1 (5.3) | 1 (5.0) | 2 (9.5) | 2 (11.1) | 1 (6.3) | 7 (7.4) | 14 (66.7) | 12 (54.5) | 12 (66.7) | 11 (61.1) | 9 (50.0) | 58 (59.8) |
| Grade 2 | 0 | 0 | 0 | 0 | 0 | 0 | 0 | 0 | 2 (9.1) | 1 (5.6) | 2 (11.1) | 5 (27.8) | 10 (10.3) |
| Grade 3 | 1 (2.0) | 0 | 0 | 0 | 1 (5.6) | 0 | 1 (1.1) | 1 (4.8) | 1 (4.5) | 1 (5.6) | 1 (5.6) | 2 (11.1) | 6 (6.2) |
| Erythema | 51 | 19 | 20 | 21 | 18 | 16 | 94 | 21 | 22 | 18 | 18 | 18 | 97 |
| Any | 0 | 0 | 0 | 0 | 0 | 0 | 0 | 0 | 1 (4.5) | 1 (5.6) | 2 (11.1) | 3 (16.7) | 7 (7.2) |
| Grade 1 | 0 | 0 | 0 | 0 | 0 | 0 | 0 | 0 | 1 (4.5) | 0 | 2 (11.1) | 1 (5.6) | 4 (4.1) |
| Grade 2 | 0 | 0 | 0 | 0 | 0 | 0 | 0 | 0 | 0 | 1 (5.6) | 0 | 2 (11.1) | 3 (3.1) |
| Grade 3 | 0 | 0 | 0 | 0 | 0 | 0 | 0 | 0 | 0 | 0 | 0 | 0 | 0 |
| Swelling | 51 | 19 | 20 | 21 | 18 | 16 | 94 | 21 | 22 | 18 | 18 | 18 | 97 |
| Any | 0 | 0 | 0 | 0 | 0 | 0 | 0 | 0 | 3 (13.6) | 0 | 2 (11.1) | 2 (11.1) | 7 (7.2) |
| Grade 1 | 0 | 0 | 0 | 0 | 0 | 0 | 0 | 0 | 3 (13.6) | 0 | 2 (11.1) | 0 | 5 (5.2) |
| Grade 2 | 0 | 0 | 0 | 0 | 0 | 0 | 0 | 0 | 0 | 0 | 0 | 2 (11.1) | 2 (2.1) |
| Grade 3 | 0 | 0 | 0 | 0 | 0 | 0 | 0 | 0 | 0 | 0 | 0 | 0 | 0 |
| Solicited systemic AR, N^c^ | 51 | 19 | 20 | 21 | 18 | 16 | 94 | 21 | 22 | 18 | 18 | 18 | 97 |
| Any solicited systemic AR | 12 (23.5) | 6 (31.6) | 10 (50.0) | 6 (28.6) | 8 (44.4) | 2 (12.5) | 32 (34.0) | 11 (52.4) | 11 (50.0) | 9 (50.0) | 15 (83.3) | 17 (94.4) | 63 (64.9) |
| Grade 1 | 8 (15.7) | 4 (21.1) | 8 (40.0) | 4 (19.0) | 5 (27.8) | 1 (6.3) | 22 (23.4) | 5 (23.8) | 2 (9.1) | 3 (16.7) | 8 (44.4) | 4 (22.2) | 22 (22.7) |
| Grade 2 | 4 (7.8) | 1 (5.3) | 2 (10.0) | 1 (4.8) | 1 (5.6) | 1 (6.3) | 6 (6.4) | 6 (28.6) | 5 (22.7) | 5 (27.8) | 3 (16.7) | 7 (38.9) | 26 (26.8) |
| Grade 3 | 0 | 1 (5.3) | 0 | 1 (4.8) | 2 (11.1) | 0 | 4 (4.3) | 0 | 4 (18.2) | 1 (5.6) | 3 (16.7) | 6 (33.3) | 14 (14.4) |
| Grade 4 | 0 | 0 | 0 | 0 | 0 | 0 | 0 | 0 | 0 | 0 | 1 (5.6) | 0 | 1 (1.0) |
| Fever | 51 | 19 | 20 | 21 | 18 | 16 | 94 | 21 | 22 | 18 | 18 | 18 | 97 |
| Any | 2 (3.9) | 0 | 0 | 0 | 0 | 0 | 0 | 0 | 3 (13.6) | 0 | 3 (16.7) | 2 (11.1) | 8 (8.2) |
| Grade 1 | 2 (3.9) | 0 | 0 | 0 | 0 | 0 | 0 | 0 | 2 (9.1) | 0 | 1 (5.6) | 1 (5.6) | 4 (4.1) |
| Grade 2 | 0 | 0 | 0 | 0 | 0 | 0 | 0 | 0 | 1 (4.5) | 0 | 0 | 1 (5.6) | 2 (2.1) |
| Grade 3 | 0 | 0 | 0 | 0 | 0 | 0 | 0 | 0 | 0 | 0 | 2 (11.1) | 0 | 2 (2.1) |
| Headache | 51 | 19 | 20 | 21 | 18 | 16 | 94 | 21 | 22 | 18 | 18 | 18 | 97 |
| Any | 7 (13.7) | 2 (10.5) | 5 (25.0) | 5 (23.8) | 5 (27.8) | 0 | 17 (18.1) | 7 (33.3) | 10 (45.5) | 8 (44.4) | 13 (72.2) | 14 (77.8) | 52 (53.6) |
| Grade 1 | 6 (11.8) | 1 (5.3) | 5 (25.0) | 4 (19.0) | 3 (16.7) | 0 | 13 (13.8) | 5 (23.8) | 5 (22.7) | 6 (33.3) | 9 (50.0) | 4 (22.2) | 29 (29.9) |
| Grade 2 | 1 (2.0) | 0 | 0 | 0 | 1 (5.6) | 0 | 1 (1.1) | 2 (9.5) | 4 (18.2) | 1 (5.6) | 3 (16.7) | 8 (44.4) | 18 (18.6) |
| Grade 3 | 0 | 1 (5.3) | 0 | 1 (4.8) | 1 (5.6) | 0 | 3 (3.2) | 0 | 1 (4.5) | 1 (5.6) | 1 (5.6) | 2 (11.1) | 5 (5.2) |
| Fatigue | 51 | 19 | 20 | 21 | 18 | 16 | 94 | 21 | 22 | 18 | 18 | 18 | 97 |
| Any | 10 (19.6) | 3 (15.8) | 5 (25.0) | 3 (14.3) | 5 (27.8) | 2 (12.5) | 18 (19.1) | 10 (47.6) | 9 (40.9) | 7 (38.9) | 12 (66.7) | 16 (88.9) | 54 (55.7) |
| Grade 1 | 6 (11.8) | 2 (10.5) | 3 (15.0) | 1 (4.8) | 4 (22.2) | 1 (6.3) | 11 (11.7) | 6 (28.6) | 1 (4.5) | 2 (11.1) | 6 (33.3) | 5 (27.8) | 20 (20.6) |
| Grade 2 | 4 (7.8) | 1 (5.3) | 2 (10.0) | 2 (9.5) | 0 | 1 (6.3) | 6 (6.4) | 4 (19.0) | 6 (27.3) | 5 (27.8) | 5 (27.8) | 8 (44.4) | 28 (28.9) |
| Grade 3 | 0 | 0 | 0 | 0 | 1 (5.6) | 0 | 1 (1.1) | 0 | 2 (9.1) | 0 | 1 (5.6) | 3 (16.7) | 6 (6.2) |
| Myalgia | 51 | 19 | 20 | 21 | 18 | 16 | 94 | 21 | 22 | 18 | 18 | 18 | 97 |
| Any | 4 (7.8) | 2 (10.5) | 3 (15.0) | 4 (19.0) | 3 (16.7) | 0 | 12 (12.8) | 6 (28.6) | 9 (40.9) | 8 (44.4) | 12 (66.7) | 14 (77.8) | 49 (50.5) |
| Grade 1 | 3 (5.9) | 1 (5.3) | 2 (10.0) | 2 (9.5) | 2 (11.1) | 0 | 7 (7.4) | 4 (19.0) | 3 (13.6) | 2 (11.1) | 5 (27.8) | 7 (38.9) | 21 (21.6) |
| Grade 2 | 1 (2.0) | 1 (5.3) | 1 (5.0) | 2 (9.5) | 0 | 0 | 4 (4.3) | 2 (9.5) | 4 (18.2) | 6 (33.3) | 6 (33.3) | 5 (27.8) | 23 (23.7) |
| Grade 3 | 0 | 0 | 0 | 0 | 1 (5.6) | 0 | 1 (1.1) | 0 | 2 (9.1) | 0 | 0 | 2 (11.1) | 4 (4.1) |
| Grade 4 | 0 | 0 | 0 | 0 | 0 | 0 | 0 | 0 | 0 | 0 | 1 (5.6) | 0 | 1 (1.0) |
| Arthralgia | 51 | 19 | 20 | 21 | 18 | 16 | 94 | 21 | 22 | 18 | 18 | 18 | 97 |
| Any | 7 (13.7) | 1 (5.3) | 3 (15.0) | 4 (19.0) | 4 (22.2) | 1 (6.3) | 13 (13.8) | 6 (28.6) | 7 (31.8) | 7 (38.9) | 8 (44.4) | 11 (61.1) | 39 (40.2) |
| Grade 1 | 6 (11.8) | 0 | 3 (15.0) | 2 (9.5) | 3 (16.7) | 1 (6.3) | 9 (9.6) | 5 (23.8) | 2 (9.1) | 1 (5.6) | 3 (16.7) | 4 (22.2) | 15 (15.5) |
| Grade 2 | 1 (2.0) | 1 (5.3) | 0 | 2 (9.5) | 1 (5.6) | 0 | 4 (4.3) | 1 (4.8) | 4 (18.2) | 6 (33.3) | 5 (27.8) | 5 (27.8) | 21 (21.6) |
| Grade 3 | 0 | 0 | 0 | 0 | 0 | 0 | 0 | 0 | 1 (4.5) | 0 | 0 | 2 (11.1) | 3 (3.1) |
| Nausea/vomiting | 51 | 19 | 20 | 21 | 18 | 16 | 94 | 21 | 22 | 18 | 18 | 18 | 97 |
| Any | 0 | 0 | 1 (5.0) | 0 | 3 (16.7) | 0 | 4 (4.3) | 1 (4.8) | 4 (18.2) | 2 (11.1) | 5 (27.8) | 3 (16.7) | 15 (15.5) |
| Grade 1 | 0 | 0 | 1 (5.0) | 0 | 2 (11.1) | 0 | 3 (3.2) | 1 (4.8) | 2 (9.1) | 1 (5.6) | 3 (16.7) | 2 (11.1) | 9 (9.3) |
| Grade 2 | 0 | 0 | 0 | 0 | 1 (5.6) | 0 | 1 (1.1) | 0 | 2 (9.1) | 1 (5.6) | 2 (11.1) | 1 (5.6) | 6 (6.2) |
| Grade 3 | 0 | 0 | 0 | 0 | 0 | 0 | 0 | 0 | 0 | 0 | 0 | 0 | 0 |
| Lymphadenopathy | 51 | 19 | 20 | 21 | 18 | 16 | 94 | 21 | 22 | 18 | 18 | 18 | 97 |
| Any | 0 | 0 | 1 (5.0) | 1 (4.8) | 0 | 0 | 2 (2.1) | 1 (4.8) | 1 (4.5) | 1 (5.6) | 1 (5.6) | 3 (16.7) | 7 (7.2) |
| Grade 1 | 0 | 0 | 1 (5.0) | 1 (4.8) | 0 | 0 | 2 (2.1) | 1 (4.8) | 0 | 0 | 1 (5.6) | 1 (5.6) | 3 (3.1) |
| Grade 2 | 0 | 0 | 0 | 0 | 0 | 0 | 0 | 0 | 1 (4.5) | 1 (5.6) | 0 | 2 (11.1) | 4 (4.1) |
| Grade 3 | 0 | 0 | 0 | 0 | 0 | 0 | 0 | 0 | 0 | 0 | 0 | 0 | 0 |
| Chills | 51 | 19 | 20 | 21 | 18 | 16 | 94 | 21 | 22 | 18 | 18 | 18 | 97 |
| Any | 3 (5.9) | 0 | 2 (10.0) | 0 | 1 (5.6) | 0 | 3 (3.2) | 3 (14.3) | 4 (18.2) | 2 (11.1) | 8 (44.4) | 12 (66.7) | 29 (29.9) |
| Grade 1 | 1 (2.0) | 0 | 1 (5.0) | 0 | 0 | 0 | 1 (1.1) | 3 (14.3) | 2 (9.1) | 2 (11.1) | 2 (11.1) | 5 (27.8) | 14 (14.4) |
| Grade 2 | 2 (3.9) | 0 | 1 (5.0) | 0 | 1 (5.6) | 0 | 2 (2.1) | 0 | 2 (9.1) | 0 | 6 (33.3) | 6 (33.3) | 14 (14.4) |
| Grade 3 | 0 | 0 | 0 | 0 | 0 | 0 | 0 | 0 | 0 | 0 | 0 | 1 (5.6) | 1 (1.0) |

Abbreviation: AR, adverse reaction.

Participants are counted only once in each category.

^b^Number of participants in the solicited safety set.

^c^Number (%) of participants in each group reporting the event, unless otherwise specified.

^d^Number of participants in the solicited safety set who submitted any data for the event.
