# Supplementary material for: Safety and Immunogenicity of an mRNA-Based RSV Vaccine Including a 12-Month Booster in a Phase 1 Clinical Trial in Healthy Older Adults
Source: J Infect Dis. 2024 Feb 22;230(3):e647–56. doi: 10.1093/infdis/jiae081 (PMC11420773; doi:10.1093/infdis/jiae081)
Supplement: jiae081_Supplementary_Data [file jiae081_supplementary_data.zip › Shaw_Supplementary_Table 6.docx]

**Table S6. Summary of Unsolicited Treatment-Emergent Adverse Events Occurring After the First Injection up to Data Cut Off (Safety Set)**

|  |  | **mRNA-1345** | | | | | |
| --- | --- | --- | --- | --- | --- | --- | --- |
|  | **Placebo** | **12.5 µg** | **25 µg** | **50 µg** | **100 µg** | **200 µg** | **Total** |
|  | **N = 59^a^** | **N = 48^b^** | **N = 48^b^** | **N = 47^b^** | **N = 48^b^** | **N = 48^b^** | **N = 239^b^** |
| n (%)^b^ |  |  |  |  |  |  |  |
| All unsolicited TEAEs | 21 (35.6) | 32 (66.7) | 24 (50.0) | 30 (63.8) | 20 (41.7) | 29 (60.4) | 135 (56.5) |
| SAEs^c^ | 1 (1.7) | 6 (12.5) | 3 (6.3) | 4 (8.5) | 0 | 1 (2.1) | 14 (5.9) |
| MAAEs | 21 (35.6) | 31 (64.6) | 24 (50.0) | 28 (59.6) | 16 (33.3) | 27 (56.3) | 126 (52.7) |
| AESIs |  |  |  |  |  |  |  |
| Thrombocytopenia | 0 | 0 | 0 | 0 | 0 | 0 | 0 |
| Myocarditis/ Pericarditis | 0 | 0 | 0 | 0 | 0 | 0 | 0 |
| Anaphylaxis | 0 | 0 | 0 | 0 | 0 | 0 | 0 |
| New onset or worsening of the neurologic diseases^d^ | 0 | 0 | 0 | 0 | 0 | 0 | 0 |
| Fatal TEAEs | 0 | 0 | 0 | 0 | 0 | 0 | 0 |
| TEAEs leading to vaccination discontinuation | 0 | 1 (2.1) | 0 | 0 | 0 | 0 | 1 (0.4) |
| TEAEs leading to study discontinuation | 0 | 0 | 0 | 0 | 0 | 0 | 0 |
| TEAEs grade ≥3 | 0 | 6 (12.5) | 3 (6.3) | 5 (10.6) | 4 (8.3) | 3 (6.3) | 21 (8.8) |
| **Treatment-related TEAEs** |  |  |  |  |  |  |  |
| All unsolicited TEAEs | 6 (10.2) | 5 (10.4) | 2 (4.2) | 2 (4.3) | 5 (10.4) | 5 (10.4) | 19 (7.9) |
| SAEs | 0 | 0 | 0 | 0 | 0 | 0 | 0 |
| MAAEs | 0 | 0 | 0 | 1 (2.1) | 0 | 0 | 1 (0.4) |
| AESIs |  |  |  |  |  |  |  |
| Thrombocytopenia | 0 | 0 | 0 | 0 | 0 | 0 | 0 |
| Myocarditis/ Pericarditis | 0 | 0 | 0 | 0 | 0 | 0 | 0 |
| Anaphylaxis | 0 | 0 | 0 | 0 | 0 | 0 | 0 |
| New onset or worsening of the neurologic diseases^d^ | 0 | 0 | 0 | 0 | 0 | 0 | 0 |
| Fatal TEAEs | 0 | 0 | 0 | 0 | 0 | 0 | 0 |
| TEAEs leading to vaccination discontinuation | 0 | 0 | 0 | 0 | 0 | 0 | 0 |
| TEAEs leading to study discontinuation | 0 | 0 | 0 | 0 | 0 | 0 | 0 |
| TEAEs grade ≥3 | 0 | 0 | 0 | 2 (4.3) | 0 | 0 | 2 (0.8) |

Abbreviations: AESI, adverse event of special interest; MAAE, medically attended adverse event; SAE, serious adverse event; TEAE, treatment-emergent adverse event.

The safety set for first vaccination consists of all randomly assigned participants who received the first study vaccine.

A TEAE was defined as any event not present before exposure to study drug or any event already present that worsened in intensity or frequency after exposure. Summaries of unsolicited TEAEs after the first injection include all TEAEs up to 28 days post first injection as well as all SAEs, MAAEs, fatal TEAEs and AESIs collected from the first injection up to the booster injection, or to end of study if participants did not receive booster injection.

^a^Number of participants in the safety set who received this vaccine.

^b^Number of participants who received this vaccine and reported the event.

^c^SAEs after Dose 1 (safety set): No SAEs were reported within 28 or 42 days after the first injection and no SAEs were considered to be related to study injection per investigator. Onset day of SAEs relative to the first injection ranged from Study Day 75 (gunshot wound) to Study Day 389. Other than 2 participants (2/239, 0.8%) with SAEs of pneumonia, both in the 12.5 µg mRNA-1345 group, no SAE by preferred term was reported for more than one participant overall.

^d^New onset or worsening of neurologic diseases include Guillain-Barré syndrome, acute disseminated encephalomyelitis, idiopathic peripheral facial nerve palsy (Bell’s palsy) and seizures, including but not limited to febrile seizures and/or generalized seizures/convulsions.
