# Supplementary material for: Safety and Immunogenicity of an mRNA-Based RSV Vaccine Including a 12-Month Booster in a Phase 1 Clinical Trial in Healthy Older Adults
Source: J Infect Dis. 2024 Feb 22;230(3):e647–56. doi: 10.1093/infdis/jiae081 (PMC11420773; doi:10.1093/infdis/jiae081)
Supplement: jiae081_Supplementary_Data [file jiae081_supplementary_data.zip › Shaw_Supplementary_Table 7.docx]

**Table S7. Summary of Unsolicited Treatment-Emergent Adverse Events After the Booster Injection up to Data Cut Off (Safety Set)**

|  |  | **mRNA-1345/Placebo** | | | | | | **mRNA-1345/mRNA-1345** | | | | | |
| --- | --- | --- | --- | --- | --- | --- | --- | --- | --- | --- | --- | --- | --- |
|  | **Placebo/**  **Placebo** | **mRNA-1345**  **12.5 µg/**  **Placebo** | **mRNA-1345**  **25 µg/**  **Placebo** | **mRNA-1345**  **50 µg/**  **Placebo** | **mRNA-1345**  **100 µg/**  **Placebo** | **mRNA-1345**  **200 µg/**  **Placebo** | **mRNA-1345/**  **Placebo Total** | **mRNA-1345**  **12.5 µg/**  **mRNA-1345 12.5 µg** | **mRNA-1345**  **25 µg/**  **mRNA-1345 25 µg** | **mRNA-1345**  **50 µg/**  **mRNA-1345 50 µg** | **mRNA-1345**  **100 µg/**  **mRNA-1345 100 µg** | **mRNA-1345**  **200 µg/**  **mRNA-1345**  **200 µg** | **mRNA-1345**  **/mRNA-1345**  **Total** |
|  | **N = 52^a^** | **N = 20^a^** | **N = 20^a^** | **N = 21^a^** | **N = 18^a^** | **N = 17^a^** | **N = 96^a^** | **N = 21^a^** | **N = 22^a^** | **N = 18^a^** | **N = 18^a^** | **N = 20^a^** | **N = 99^a^** |
| **After first Injection, n (%)^b^** | | | | | | |  |  |  |  |  |  |  |
| All Unsolicited TEAEs | 20 (38.5) | 14 (70.0) | 11 55.0) | 11 (52.4) | 8 (44.4) | 10 (58.8) | 54 (56.3) | 13 (61.9) | 11 (50.0) | 13 (72.2) | 8 (44.4) | 12 (60.0) | 57 (57.6) |
| SAEs | 1 (1.9) | 1 (5.0) | 1 (5.0) | 1 (4.8) | 0 | 0 | 3 (3.1) | 2 (9.5) | 1 (4.5) | 2 (11.1) | 0 | 0 | 5 (5.1) |
| MAAEs | 20 (38.5) | 14 (70.0) | 11 (55.0) | 11 (52.4) | 7 (38.9) | 10 (58.8) | 53 (55.2) | 12 (57.1) | 11 (50.0) | 12 (66.7) | 6 (33.3) | 11 (55.0) | 52 (52.5) |
| AESIs |  |  |  |  |  |  |  |  |  |  |  |  |  |
| Thrombocytopenia | 0 | 0 | 0 | 0 | 0 | 0 | 0 | 0 | 0 | 0 | 0 | 0 | 0 |
| Myocarditis/Pericarditis | 0 | 0 | 0 | 0 | 0 | 0 | 0 | 0 | 0 | 0 | 0 | 0 | 0 |
| Anaphylaxis | 0 | 0 | 0 | 0 | 0 | 0 | 0 | 0 | 0 | 0 | 0 | 0 | 0 |
| New onset or worsening of the neurologic diseases^c^ | 0 | 0 | 0 | 0 | 0 | 0 | 0 | 0 | 0 | 0 | 0 | 0 | 0 |
| Fatal TEAEs | 0 | 0 | 0 | 0 | 0 | 0 | 0 | 0 | 0 | 0 | 0 | 0 | 0 |
| TEAEs leading to vaccination discontinuation | 0 | 0 | 0 | 0 | 0 | 0 | 0 | 0 | 0 | 0 | 0 | 0 | 0 |
| TEAEs leading to study discontinuation | 0 | 0 | 0 | 0 | 0 | 0 | 0 | 0 | 0 | 0 | 0 | 0 | 0 |
| TEAEs grade ≥3 | 0 | 1 (5.0) | 1 (5.0) | 2 (9.5) | 1 (5.6) | 1 (5.9) | 6 (6.3) | 2 (9.5) | 1 (4.5) | 2 (11.1) | 2 (11.1) | 0 | 7 (7.1) |
| **Treatment-related TEAEs** |  |  |  |  |  |  |  |  |  |  |  |  |  |
| All Unsolicited TEAEs | 6 (11.5) | 2 (10.0) | 2 (10.0) | 2 (9.5) | 0 | 1 (5.9) | 7 (7.3) | 1 (4.8) | 0 | 0 | 3 (16.7) | 2 (10.0) | 6 (6.1) |
| SAEs | 0 | 0 | 0 | 0 | 0 | 0 | 0 | 0 | 0 | 0 | 0 | 0 | 0 |
| MAAEs | 0 | 0 | 0 | 1 (4.8) | 0 | 0 | 1 (1.0) | 0 | 0 | 0 | 0 | 0 | 0 |
| AESIs |  |  |  |  |  |  |  |  |  |  |  |  |  |
| Thrombocytopenia | 0 | 0 | 0 | 0 | 0 | 0 | 0 | 0 | 0 | 0 | 0 | 0 | 0 |
| Myocarditis/Pericarditis | 0 | 0 | 0 | 0 | 0 | 0 | 0 | 0 | 0 | 0 | 0 | 0 | 0 |
| Anaphylaxis | 0 | 0 | 0 | 0 | 0 | 0 | 0 | 0 | 0 | 0 | 0 | 0 | 0 |
| New onset or worsening of the neurologic diseases^c^ | 0 | 0 | 0 | 0 | 0 | 0 | 0 | 0 | 0 | 0 | 0 | 0 | 0 |
| Fatal TEAEs | 0 | 0 | 0 | 0 | 0 | 0 | 0 | 0 | 0 | 0 | 0 | 0 | 0 |
| TEAEs leading to vaccination discontinuation | 0 | 0 | 0 | 0 | 0 | 0 | 0 | 0 | 0 | 0 | 0 | 0 | 0 |
| TEAEs leading to study discontinuation | 0 | 0 | 0 | 0 | 0 | 0 | 0 | 0 | 0 | 0 | 0 | 0 | 0 |
| TEAEs grade ≥3 | 0 | 0 | 0 | 2 (9.5) | 0 | 0 | 2 (2.1) | 0 | 0 | 0 | 0 | 0 | 0 |
| **After Booster Injection, n (%)^b^** | | |  |  |  |  |  | |  |  |  |  |  |
| All Unsolicited TEAEs | 10 (19.2) | 10 (50.0) | 6 (30.0) | 4 (19.0) | 2 (11.1) | 4 (23.5) | 26 (27.1) | 4 (19.0) | 9 (40.9) | 3 (16.7) | 3 (16.7) | 7 (35.0) | 26 (26.3) |
| SAEs | 1 (1.9) | 1 (5.0) | 1 (5.0) | 1 (4.8) | 0 | 1 (5.9) | 4 (4.2) | 0 | 2 (9.1) | 0 | 1 (5.6) | 0 | 3 (3.0) |
| MAAEs | 8 (15.4) | 6 (30.0) | 3 (15.0) | 3 (14.3) | 2 (11.1) | 3 (17.6) | 17 (17.7) | 4 (19.0) | 6 (27.3) | 2 (11.1) | 2 (11.1) | 6 (30.0) | 20 (20.2) |
| AESIs |  |  |  |  |  |  |  |  |  |  |  |  |  |
| Thrombocytopenia | 0 | 0 | 0 | 0 | 0 | 0 | 0 | 0 | 0 | 0 | 0 | 0 | 0 |
| Myocarditis/ Pericarditis | 0 | 0 | 0 | 0 | 0 | 0 | 0 | 0 | 0 | 0 | 0 | 0 | 0 |
| Anaphylaxis | 0 | 0 | 0 | 0 | 0 | 0 | 0 | 0 | 0 | 0 | 0 | 0 | 0 |
| New onset or worsening of the neurologic diseases^c^ | 0 | 0 | 0 | 0 | 0 | 0 | 0 | 0 | 0 | 0 | 0 | 0 | 0 |
| Fatal TEAEs^d^ | 0 | 1 (5.0) | 1 (5.0) | 0 | 0 | 0 | 2 (2.1) | 0 | 0 | 0 | 0 | 0 | 0 |
| TEAEs leading to vaccination discontinuation | 0 | 0 | 0 | 0 | 0 | 0 | 0 | 0 | 0 | 0 | 0 | 0 | 0 |
| TEAEs leading to study discontinuation | 0 | 0 | 0 | 0 | 0 | 0 | 0 | 0 | 0 | 0 | 0 | 0 | 0 |
| TEAEs grade ≥3 | 1 (1.9) | 1 (5.0) | 1 (5.0) | 1 (4.8) | 0 | 1 (5.9) | 4 (4.2) | 0 | 2 (9.1) | 0 | 1 (5.6) | 0 | 3 (3.0) |
| **Treatment-related TEAEs** |  |  |  |  |  |  |  |  |  |  |  |  |  |
| All Unsolicited TEAEs | 2 (3.8) | 1 (5.0) | 2 (10.0) | 0 | 0 | 0 | 3 (3.1) | 0 | 1 (4.5) | 1 (5.6) | 1 (5.6) | 2 (10.0) | 5 (5.1) |
| SAEs | 0 | 0 |  | 0 | 0 | 0 |  | 0 | 0 | 0 | 0 | 0 | 0 |
| MAAEs^e^ | 0 | 0 | 1 (5.0) | 0 | 0 | 0 | 1 (1.0) | 0 | 0 | 0 | 0 | 1 (5.0) | 1 (1.0) |
| AESIs |  |  |  |  |  |  |  |  |  |  |  |  |  |
| Thrombocytopenia | 0 | 0 | 0 | 0 | 0 | 0 | 0 | 0 | 0 | 0 | 0 | 0 | 0 |
| Myocarditis/Pericarditis | 0 | 0 | 0 | 0 | 0 | 0 | 0 | 0 | 0 | 0 | 0 | 0 | 0 |
| Anaphylaxis | 0 | 0 | 0 | 0 | 0 | 0 | 0 | 0 | 0 | 0 | 0 | 0 | 0 |
| New onset or worsening of the neurologic diseases^c^ | 0 | 0 | 0 | 0 | 0 | 0 | 0 | 0 | 0 | 0 | 0 | 0 | 0 |
| Fatal TEAEs | 0 | 0 | 0 | 0 | 0 | 0 | 0 | 0 | 0 | 0 | 0 | 0 | 0 |
| TEAEs leading to vaccination discontinuation | 0 | 0 | 0 | 0 | 0 | 0 | 0 | 0 | 0 | 0 | 0 | 0 | 0 |
| TEAEs leading to study discontinuation | 0 | 0 | 0 | 0 | 0 | 0 | 0 | 0 | 0 | 0 | 0 | 0 | 0 |
| TEAEs grade ≥3 | 0 | 0 | 0 | 0 | 0 | 0 | 0 | 0 | 0 | 0 | 0 | 0 | 0 |

Abbreviations: AESI, adverse event of special interest; MAAE, medically attended adverse event; SAE, serious adverse event; TEAE, treatment-emergent adverse event.

The safety set for the booster vaccination consists of all randomly assigned participants who received both first and booster study vaccination.

A TEAE was defined as any event not present before exposure to study drug or any event already present that worsened in intensity or frequency after exposure. Summaries of unsolicited TEAEs after the first injection include all TEAEs up to 28 days post first injection as well as all SAEs, MAAEs, fatal TEAEs and AESIs collected from the first injection up to the booster injection. Summaries of unsolicited TEAEs after the booster injection include all TEAEs up to 28 days post the booster injection, plus all MAAEs and AESIs collected from the booster injection up to Month 14 as well as all SAEs and fatal TEAEs collected from the booster injection until the database lock date (6 February 2023).

^a^Number of participants in the safety set who received this vaccine.

^b^Number of participants who received this vaccine and reported the event.

^c^New onset or worsening of neurologic diseases include Guillain-Barré syndrome, acute disseminated encephalomyelitis, idiopathic peripheral facial nerve palsy (Bell’s palsy) and seizures, including but not limited to febrile seizures and/or generalized seizures/convulsions.

^d^A recipient of mRNA-1345 12.5 μg/placebo died due to bone sarcoma and a recipient of mRNA-1345 25 μg/placebo died due to a road traffic accident; both deaths were reported >28 days after the 12-month placebo injection and >1 year after the first mRNA-1345 injection.

^e^No serious related MAAEs within 28 or 42 days were reported. Non-serious related MAAEs per investigator assessment within 28 days included the following: A female participant aged 73 years experienced a related MAAE of dehydration and an unrelated MAAE of urinary tract infection on Day 2 after booster injection. Dehydration resolved the same day. The site did not report an end date for urinary tract infection. A participant aged 70 years experienced fatigue and arthralgia, which were collected as a solicited ARs that began on Day 3 and ended on Day 12. Solicited ARs that went beyond Day 8 were also recorded on the AE log.
