# Supplementary material for: Safety and Immunogenicity of an mRNA-Based RSV Vaccine Including a 12-Month Booster in a Phase 1 Clinical Trial in Healthy Older Adults
Source: J Infect Dis. 2024 Feb 22;230(3):e647–56. doi: 10.1093/infdis/jiae081 (PMC11420773; doi:10.1093/infdis/jiae081)
Supplement: jiae081_Supplementary_Data [file jiae081_supplementary_data.zip › Shaw_Supplementary_Table 8.docx]

**Table S8. Neutralizing Antibody Titers After the First Injection (Per-protocol Set)**

|  | **Placebo** |  |  | **mRNA-1345** |  |  |
| --- | --- | --- | --- | --- | --- | --- |
|  | **N = 58^a^** | **12.5 µg**  **N = 46^a^** | **25 µg**  **N = 46^a^** | **50 µg**  **N = 47^a^** | **100 µg**  **N = 46^a^** | **200 µg**  **N = 47^a^** |
| **RSV-A–Neutralizing Antibodies (IU/mL)** | | | | | | |
| Baseline (day 1), n^b^ | 58 | 46 | 46 | 47 | 46 | 47 |
| GMT (95% CI) | 1590.7  (1141.8, 2215.9) | 1329.8  (969.1, 1824.8) | 1519.0  (1128.8, 2044.0) | 1204.7  (918.5, 1580.0) | 1224.9  (877.7, 1709.4) | 1879.9  (1403.5, 2517.9) |
| Month 1 (day 29), n^c^ | 56 | 44 | 45 | 44 | 43 | 47 |
| GMT (95% CI) | 1827.2  (1306.1, 2556.2) | 13 619.5  (9340.7, 19 858.3) | 19 008.4  (14 470.5, 24 969.5) | 13 739.0  (9875.5, 19 113.8) | 17 053.4  (12 486.8, 23 289.9) | 31 084.4  (24 302.8, 39 758.5) |
| GMFR (95% CI) | 1.15  (0.99, 1.34) | 10.19  (7.17, 14.48) | 12.17  (8.90, 16.64) | 12.03  (8.78, 16.47) | 14.14  (10.23, 19.54) | 16.54  (12.25, 22.33) |
| Month 2 (day 57), n^c^ | 58 | 44 | 42 | 46 | 39 | 44 |
| GMT (95% CI) | 1783.9  (1257.5, 2530.7) | 8958.6  (6238.2, 12 865.4) | 15 115.3  (10 704.5, 21 343.6) | 10 967.5  (7762.9, 15 494.9) | 11 858.0  (8445.9, 16 648.4) | 24 289.2  (18 725.5, 31506.1) |
| GMFR (95% CI) | 1.12  (0.95, 1.33) | 6.61  (4.92, 8.89) | 10.03  (7.17, 14.02) | 9.16  (6.73, 12.47) | 9.31  (6.65, 13.04) | 12.70  (9.53, 16.92) |
| Month 3 (day 85), n^c^ | 55 | 43 | 45 | 44 | 42 | 46 |
| GMT (95% CI) | 1761.0  (1243.8, 2493.3) | 7192.1  (4987.4, 10 371.4) | 11 372.8  (8253.5, 15671.1) | 9020.8  (6523.6, 12 474.0) | 9947.3  (7262.5, 13 624.5) | 17624.2  (13 033.4, 23832.0) |
| GMFR (95% CI) | 1.11  (0.98, 1.26) | 5.27  (3.82, 7.29) | 7.54  (5.57, 10.22) | 7.53  (5.54, 10.24) | 7.73  (5.95, 10.05) | 9.35  (6.76, 12.92) |
| Month 6 (day 169), n^c^ | 54 | 44 | 44 | 43 | 42 | 44 |
| GMT (95% CI) | 1636.4  (1167.8, 2293.2) | 4263.2  (3028.4, 6001.6) | 5936.5  (4280.0, 8234.3) | 5746.9  (4133.8, 7989.4) | 4815.2  (3517.0, 6592.4) | 10 580.9  (7747.7, 14450.1) |
| GMFR (95% CI) | 1.00  (0.87, 1.17) | 3.08  (2.33, 4.08) | 4.10  (3.04, 5.54) | 5.05  (3.77, 6.76) | 4.05  (2.96, 5.54) | 5.74  (4.54, 7.25) |
| Month 12 (day 365), n^c^ | 49 | 40 | 41 | 39 | 33 | 37 |
| GMT (95% CI) | 2035.5  (1403.1, 2952.8) | 3161.4  (2187.0, 4570.0) | 4592.4  (3343.7, 6307.5) | 3531.9  (2376.0, 5250.2) | 2525.7  (1795.9, 3551.9) | 6201.2  (4251.6, 9044.7) |
| GMFR (95% CI) | 1.15  (0.96, 1.38) | 2.39  (1.84, 3.10) | 2.96  (2.22, 3.94) | 3.00  (2.18, 4.13) | 2.66  (1.94, 3.66) | 3.16  (2.38, 4.20) |
| **RSV-B–Neutralizing Antibodies (IU/mL)** | | | | | | |
| Baseline (day 1), n^b^ | 58 | 46 | 46 | 47 | 46 | 47 |
| GMT (95% CI) | 1450.8  (1053.2, 1998.7) | 1437.5  (1015.1, 2035.4) | 1507.7  (1055.3, 2153.9) | 1135.3  (833.2, 1547.0) | 941.0  (681.6, 1299.1) | 1455.4  (1008.2, 2100.9) |
| Month 1 (day 29), n^c^ | 56 | 44 | 45 | 44 | 43 | 47 |
| GMT (95% CI) | 1579.9  (1102.0, 2265.2) | 8154.1  (5568.1, 11941.1) | 10 235.2  (7445.9, 14069.5) | 9432.1  (6706.2, 13 266.0) | 9319.9  (6754.5, 12 859.7) | 18 183.8  (13 206.2, 25 037.5) |
| GMFR (95% CI) | 1.12  (0.98, 1.29) | 5.29  (3.74, 7.49) | 6.56  (4.86, 8.87) | 8.96  (6.79, 11.84) | 9.60  (7.31, 12.61) | 12.49  (9.10, 17.16) |
| Month 2 (day 57), n^c^ | 58 | 44 | 42 | 46 | 39 | 44 |
| GMT (95% CI) | 1840.6  (1332.2, 2543.1) | 5864.7  (4178.3, 8231.8) | 8004.0  (5684.9, 11269.1) | 7905.3  (5765.3, 10 839.5) | 8869.0  (6455.4, 12 185.0) | 15 182.3  (11 431.9, 20 163.0) |
| GMFR (95% CI) | 1.27  (1.12, 1.44) | 4.02  (3.08, 5.23) | 5.66  (4.35, 7.36) | 7.08  (5.40, 9.28) | 8.87  (6.71, 11.73) | 9.77  (7.34, 13.00) |
| Month 3 (day 85), n^c^ | 55 | 43 | 45 | 44 | 42 | 46 |
| GMT (95% CI) | 1874.2  (1375.7, 2553.3) | 5065.2  (3432.3, 7474.8) | 6754.9  (4823.0, 9460.6) | 6385.1  (4652.7, 8762.4) | 6931.0  (4873.8, 9856.6) | 11 984.5  (8513.3, 16 871.1) |
| GMFR (95% CI) | 1.27  (1.12, 1.44) | 3.51  (2.58, 4.78) | 4.57  (3.54, 5.90) | 5.62  (4.24, 7.45) | 7.02  (5.35, 9.20) | 7.99  (6.00, 10.64) |
| Month 6 (day 169), n^c^ | 54 | 44 | 44 | 43 | 42 | 44 |
| GMT (95% CI) | 1957.0  (1433.4, 2671.8) | 4164.0  (2876.3, 6028.0) | 4873.5  (3518.5, 6750.3) | 5226.3  (3747.5, 7288.8) | 4313.9  (3166.9, 5876.2) | 8171.7  (6023.4, 11 086.3) |
| GMFR (95% CI) | 1.35  (1.16, 1.57) | 2.87  (2.24, 3.70) | 3.20  (2.36, 4.34) | 4.38  (3.37, 5.70) | 4.60  (3.69, 5.73) | 5.50  (4.39, 6.89) |
| Month 12 (day 365), n^c^ | 49 | 40 | 41 | 39 | 33 | 37 |
| GMT (95% CI) | 1673.2  (1190.9, 2350.8) | 2113.9  (1548.6, 2885.6) | 2321.2  (1657.2, 3251.1) | 2590.2  (1868.6, 3590.3) | 2475.8  (1791.4, 3421.5) | 4127.4  (2998.1, 5682.1) |
| GMFR (95% CI) | 1.07  (0.89, 1.29) | 1.52  (1.23, 1.88) | 1.61  (1.25, 2.06) | 2.27  (1.77, 2.91) | 2.78  (2.10, 3.69) | 2.92  (2.32, 3.68) |

Abbreviations: CI, confidence interval; IU, international units; GMT, geometric mean titer; GMFR, geometric mean fold-rise, comparing post-baseline to

baseline titer values; LLOQ, lower limit of quantitation; RSV, respiratory syncytial virus; ULOQ, upper limit of quantitation.

95% CI was calculated based on the t-distribution of the log-transformed values for GMT, then back-transformed to the original scale for presentation; 95% CI for other measures were calculated using the Clopper-Pearson method.

For geometric mean fold rise (GMFR), comparing post-baseline to baseline titer values, antibody values reported as below LLOQ at baseline were replaced by LLOQ.

For GMT and GMC calculations, antibody values reported as below LLOQ were replaced by 0.5 × LLOQ.

RSV-A (IU/mL): LLOQ = 11, ULOQ = 176 050.

RSV-B (IU/mL): LLOQ = 8, ULOQ = 111 998.

^a^Number of participants in any per-protocol set.

^b^Number of participants with nonmissing baseline data.

^c^Number of participants with nonmissing data in the corresponding category at the corresponding time point.
