# Supplementary material for: Safety and Immunogenicity of an mRNA-Based RSV Vaccine Including a 12-Month Booster in a Phase 1 Clinical Trial in Healthy Older Adults
Source: J Infect Dis. 2024 Feb 22;230(3):e647–56. doi: 10.1093/infdis/jiae081 (PMC11420773; doi:10.1093/infdis/jiae081)
Supplement: jiae081_Supplementary_Data [file jiae081_supplementary_data.zip › Shaw_Supplementary_Table 9.docx]

**Table S9. Binding Antibody Concentrations After the First Injection (Per-protocol Set)**

|  | **Placebo** |  |  | **mRNA-1345** |  |  |
| --- | --- | --- | --- | --- | --- | --- |
|  | **N = 58^a^** | **12.5 µg**  **N = 46^a^** | **25 µg**  **N = 46^a^** | **50 µg**  **N = 47^a^** | **100 µg**  **N = 46^a^** | **200 µg**  **N = 47^a^** |
| **PreF-Binding Antibodies (AU/mL)** | | | | | | |
| Baseline (day 1), n^b^ | 58 | 46 | 46 | 47 | 46 | 47 |
| GMC (95% CI) | 7871.3  (6388.4, 9698.5) | 8077.0  (6430.5, 10 145.0) | 9134.1  (7414.4, 11 252.7) | 7184.6  (5872.3, 8790.1) | 6960.5  (5456.3, 8879.3) | 9173.2  (7267.6, 11 578.5) |
| Month 1 (day 29), n^c^ | 56 | 44 | 45 | 44 | 43 | 47 |
| GMC (95% CI) | 8119.5  (6379.3, 10 334.4) | 59 833.7  (45 903.3, 77 991.6) | 77 569.9  (62 867.0, 95 711.4) | 58 206.6  (46 911.4, 72 221.3) | 80 760.1  (67 929.5, 96 014.0) | 111 145.6  (87 974.7, 140 419.4) |
| GMFR (95% CI) | 1.0  (0.9, 1.2) | 7.2  (5.6, 9.3) | 8.4  (6.8, 10.4) | 8.5  (6.8, 10.6) | 11.6  (9.2, 14.6) | 12.1  (9.2, 16.0) |
| Month 2 (day 57), n^c^ | 58 | 44 | 42 | 46 | 39 | 44 |
| GMC (95% CI) | 8164.6  (6555.1, 10 169.3) | 39 632.4  (30 681.8, 51 194.1) | 56 901.4  (44 316.0, 73 060.9) | 50 630.1  (41 214.3, 62 197.1) | 56 860.8  (47 987.0, 67 375.5) | 85 785.4  (70 699.4, 104 090.5) |
| GMFR (95% CI) | 1.0  (0.9, 1.2) | 4.9  (3.9, 6.2) | 6.6  (5.3, 8.2) | 7.0  (5.8, 8.6) | 7.8  (6.3, 9.7) | 9.3  (7.2, 11.9) |
| Month 3 (day 85), n^c^ | 55 | 43 | 45 | 44 | 42 | 46 |
| GMC (95% CI) | 8157.9  (6427.1, 10 354.9) | 35 150.3  (26 811.4, 46 082.7) | 48 550.6  (38 678.9, 60 941.8) | 39 390.5  (31 889.7, 48 655.6) | 47 141.3  (40 078.1, 55 449.3) | 64 628.3  (52 007.5, 80 311.8) |
| GMFR (95% CI) | 1.0  (0.9, 1.1) | 4.3  (3.4, 5.5) | 5.4  (4.4, 6.6) | 5.4  (4.3, 6.9) | 6.5  (5.3, 8.1) | 7.0  (5.4, 8.9) |
| Month 6 (day 169), n^c^ | 54 | 44 | 44 | 43 | 42 | 44 |
| GMC (95% CI) | 8321.7  (6676.1, 10 372.8) | 23 376.4  (18 019.0, 30 326.6) | 29 704.2  (23 626.6, 37 345.1) | 27 977.0  (22 479.6, 34 818.8) | 27 948.6  (22 579.4, 34 594.4) | 41 137.0  (33 523.9, 50 479.0) |
| GMFR (95% CI) | 1.0  (0.9, 1.2) | 2.8  (2.3, 3.5) | 3.3  (2.7, 4.1) | 4.0  (3.3, 4.9) | 4.1  (3.3, 5.1) | 4.7  (3.8, 5.8) |
| Month 12 (day 365), n^c^ | 49 | 40 | 41 | 39 | 33 | 37 |
| GMC (95% CI) | 9660.9  (7556.6, 12 351.2) | 16 715.1  (12 878.1, 21 695.2) | 21 454.1  (17 263.3, 26 662.3) | 19 404.6  (15 117.4, 24 907.7) | 19 632.9  (15 902.1, 24 238.9) | 30 954.5  (24 140.5, 39 691.9) |
| GMFR (95% CI) | 1.2  (1.0, 1.4) | 2.1  (1.7, 2.6) | 2.4  (2.0, 3.0) | 2.8  (2.3, 3.4) | 3.4  (2.6, 4.3) | 3.3  (2.6, 4.1) |
| **PostF-Binding Antibodies (AU/mL)** | | | | | | |
| Baseline (day 1), n^b^ | 58 | 46 | 46 | 47 | 46 | 47 |
| GMC (95% CI) | 11 806.0  (9501.6, 14 669.2) | 10 454.3  (7820.9, 13 974.4) | 14 206.6  (10 853.0, 18 596.5) | 12 066.0  (9323.5, 15 615.2) | 10 638.2  (8135.1, 13 911.5) | 10 424.6  (8167.5, 13 305.5) |
| Month 1 (day 29), n^c^ | 56 | 44 | 45 | 44 | 43 | 47 |
| GMC (95% CI) | 12 067.1 (9309.9, 15 640.8) | 47 736.3 (35 493.9, 64 201.5) | 84 909.7 (64 905.4, 111 079.5) | 69 105.0 (52 552.2, 90 871.5) | 85 033.1 (66619.6, 108535.9) | 95 879.2  (71829.4, 127981.4) |
| GMFR (95% CI) | 1.0  (0.9, 1.2) | 4.4  (3.6, 5.3) | 5.9  (4.5, 7.6) | 5.9  (4.6, 7.5) | 7.9  (6.2, 10.0) | 9.2  (7.1, 11.9) |
| Month 2 (day 57), n^c^ | 58 | 44 | 42 | 46 | 39 | 44 |
| GMC (95% CI) | 12 029.2  (9463.0, 15 291.4) | 36 081.1 (26 969.7, 48 270.7) | 59 943.4 (43 861.2, 81 922.2) | 57 804.6 (45 307.2, 73 749.2) | 51 619.0 (40 747.4, 65 391.2) | 78 968.1 (61 971.7, 100 626.0) |
| GMFR (95% CI) | 1.0  (0.9, 1.1) | 3.4  (2.8, 4.1) | 4.1  (3.1, 5.5) | 4.9  (3.9, 6.0) | 5.0  (4.0, 6.2) | 7.4  (5.9, 9.3) |
| Month 3 (day 85), n^c^ | 55 | 43 | 45 | 44 | 42 | 46 |
| GMC (95% CI) | 12 343.8  (9609.4, 15 856.1) | 30 765.7  (22 703.4, 41 691.1) | 54 054.5 (40 304.3, 72 495.8) | 45 084.0 (34 300.5, 59 257.6) | 48 819.9 (39 107.1, 60 945.1) | 59 983.2 (45 895.2, 78 395.6) |
| GMFR (95% CI) | 1.0  (0.9, 1.1) | 2.9  (2.4, 3.4) | 3.8  (3.0, 4.9) | 3.8  (2.8, 5.0) | 4.3  (3.4, 5.4) | 5.7  (4.5, 7.1) |
| Month 6 (day 169), n^c^ | 54 | 44 | 44 | 43 | 42 | 44 |
| GMC (95% CI) | 12 229.4 (9744.3, 15 348.3) | 20 712.5 (15 371.7, 27 908.9) | 37 080.4  (27 865.2, 49 343.2) | 35 691.0 (27 328.8, 46 611.8) | 29 608.9 (22 904.2, 38 276.1) | 39 154.9 (30 112.5, 50 912.6) |
| GMFR (95% CI) | 1.0  (0.9, 1.1) | 1.9  (1.7, 2.3) | 2.6  (2.0, 3.3) | 3.0  (2.5, 3.6) | 2.7  (2.2, 3.4) | 3.8  (3.1, 4.6) |
| Month 12 (day 365), n^c^ | 49 | 40 | 41 | 39 | 33 | 37 |
| GMC (95% CI) | 13 768.8  (10 464.4, 18 116.6) | 16 381.1  (11 727.9, 22 880.5) | 26 193.9  (19 873.3, 34 524.7) | 25 574.7  (19 190.7, 34 082.5) | 21 589.3  (16 517.4, 28 218.6) | 27 833.1  (20 408.2, 37 959.5) |
| GMFR (95% CI) | 1.2  (1.0, 1.4) | 1.6  (1.4, 1.8) | 1.9  (1.5, 2.4) | 2.2  (1.8, 2.7) | 2.3  (1.9, 2.9) | 2.7  (2.2, 3.2) |

Abbreviations: AU, arbitrary units; bAb, binding antibody; CI, confidence interval; GMC, geometric mean concentrations; GMFR, geometric mean fold-rise, comparing postbaseline to baseline titer values; LLOQ, lower limit of quantitation; PreF, prefusion; ULOQ, upper limit of quantitation.

95% CI is calculated based on the t-distribution of the log-transformed values for GMC, then back-transformed to the original scale for presentation; 95% CI for other measures are calculated using the Clopper-Pearson method.

For GMFR, comparing post-baseline to baseline titer values, antibody values reported as below LLOQ at baseline were replaced by LLOQ.

For GMC calculations, antibody values reported as below LLOQ were replaced by 0.5 × LLOQ.

PreF-binding antibody: LLOQ = 19.00 (AU/mL), ULOQ = 1 111 100 (AU/mL).

PostF-binding antibody: LLOQ = 16.00 (AU/mL), ULOQ = 1 111 100 (AU/mL).

^a^Number of participants in any per-protocol set.

^b^Number of participants with nonmissing baseline data.

^c^Number of participants with nonmissing data in the corresponding category at the corresponding time point.
